# To Punish or to Restore: How Children Evaluate Victims' Responses to Immorality

**DOI:** 10.3389/fpsyg.2021.696160

**Published:** 2021-08-13

**Authors:** Xin Liu, Xin Yang, Zhen Wu

**Affiliations:** ^1^Department of Psychology, Tsinghua University, Beijing, China; ^2^Department of Psychology, Yale University, New Haven, CT, United States

**Keywords:** moral evaluation, restorative justice, retributive justice, degree of punishment, possession violation

## Abstract

Punishment is important for deterring transgressions and maintaining cooperation, while restoration is also an effective way to resolve conflicts and undo harm. Which way do children prefer when evaluating others' reactions to immorality? Across four experiments, Chinese preschoolers (aged 4–6, *n* = 184) evaluated victims' different reactions to possession violations (i.e., punishing the perpetrator or restoring the belongings). Children evaluated restorative reactions more positively than punitive ones. This tendency to favor restoration over punishment was influenced by the degree of punishment, with more pronounced patterns observed when punishment was harsher (Experiments 1–3). Indeed, when different degrees of punishment were directly contrasted (Experiment 4), children viewed victims who imposed milder punishment (“steal one object, remove one or two objects”) more positively than those who imposed harsh punishment (“steal one object, remove three objects”). These patterns were especially manifested in preschoolers who chose restoration when being put in the victim's situation, suggesting a consistency between evaluations and behaviors. Taken together, the current study showed that children prioritize protecting the victim over harshly punishing the perpetrator, which suggests an early take on the preferred way to uphold justice.

## Introduction

Across different cultures, justice is one of the most crucial positive virtues (Peterson and Seligman, 2004). Converging theories and empirical evidence suggest that justice has evolved in the ecological context of pressure to maintain cooperation (Tyler, 2009), positive social interactions (Cohen, 1991), and social norms (Boyles et al., 2008). Other theories in the interdisciplinary field (e.g., mathematics, Capraro and Perc, 2021; physics, Perc, 2016) have also emphasized the importance of justice and cooperation. Justice ensures that people receive the benefits and punishment they deserve. For example, when facing possession violations, one may return objects to their rightful owners and (or) punish the perpetrator to a fair degree. Both solutions sustained justice. Traditionally, studies focus on how people enforce justice through punishment (e.g., Henrich et al., 2006). However, recent studies reveal that compared to punishment, people prefer to compensate victims and restore the possessions when these options are available (e.g., FeldmanHall et al., 2014; Riedl et al., 2015; Yang et al., 2021). The debate over the priority of punishment vs. restoration touches on principles we use when dealing with injustice. The current study approaches this debate from a developmental perspective: how do children evaluate victims' different responses to possession violations, such as punishing the perpetrator or returning the possessions to the victim? Studying young children's preferences may provide hints at human nature in upholding justice.

Punishment has traditionally been defined as a penalty or retribution directed toward those who cause harm or violate social norms (Clutton-Brock and Parker, 1995). In fairness violation (Fowler, 2005; Herrmann et al., 2008) and situations that ask for rehabilitating justice (e.g., illegality and crime, Heffner and FeldmanHall, 2019), people show the desire for punishment. Punishment is the common method in the judicial system that is widespread across human societies and plays an important role in ensuring social harmony (Hofmann et al., 2018). It serves as a powerful tool to support the cooperative system by deterring selfishness, decreasing incentives that take advantage of the system, and rewarding behaviors that comply with norms in the long run (Fehr and Gächter, 2002; Krasnow et al., 2016). It may also be used for reputational reasons, as people are more likely to punish norm violations when observed (Kurzban et al., 2007); moreover, those who have enacted punishment are judged as more trustworthy (Jordan et al., 2016). The preference for punishment emerges early in development. Infants as young as 6 month prefer individuals who act negatively toward antisocial others (Hamlin et al., 2011; Kanakogi et al., 2017). Three-year-olds punish selfish peers both when they are directly affected (Wu and Gao, 2018) and when they are third-party observers (Vaish et al., 2011). At 6 years of age, children take a cost to punish selfish peers even as unaffected third-party observers (McAuliffe et al., 2015; Salali et al., 2015). In addition, 5-year-olds allocate unpleasant items to antisocial adults anonymously (Kenward and Osth, 2015) and choose to play with a character who shows retaliating aggression to the perpetrator, again implying a preference for punishment (Etchu, 2005). These findings all speak to the possibility that children may positively evaluate punitive behaviors and victims who punish perpetrators.

Despite the well-documented evidence on the preference for punishment (e.g., Henrich et al., 2006), it is important to note that past studies usually contrast punishment with “inaction”—doing nothing or accepting the injustice (e.g., McAuliffe et al., 2015; Wu and Gao, 2018). Therefore, it is unclear whether children prefer punishment, or they merely dislike “doing nothing” in the face of injustice. Indeed, recent work has revealed that when alternative actions are available, punishment is not always the preferred way to resolve conflict. For example, when facing property loss or unfair distributions, adults prefer to compensate the victims rather than punish the perpetrators (e.g., Lotz et al., 2011; FeldmanHall et al., 2014; Heffner and FeldmanHall, 2019). These findings suggest that restorative actions may be a preferred avenue to restore justice at least in adults. In different fields of social science such as law and criminology, scholars have argued that restoration, as compared to punishment, calls attention to victim's welfare (Wenzel et al., 2008). Restoration is also beneficial for repairing the relationship destroyed by the perpetrator, thereby maintaining cooperation (McCullough, 2008).

Recent developmental work with both punishment and restoration options also shows a similar preference for restoration in children. In face of the unpermitted loss of their own or others' possessions, children from age three choose to intervene by returning the possessions to the original owner (restoration) rather than by removing the possessions to a place inaccessible to the perpetrator (punishment) (Riedl et al., 2015; Yang et al., 2021). In a separate line of work, 5–9-year-olds prefer third-party helping to third-party punishment (Lee and Warneken, 2020). These findings suggest that punishment is not always favored by children, and other options such as restoration is sometimes more valued.

However, open questions remain concerning children's preference between restoration and punishment. To begin with, the developmental work reviewed above examined either children's own behaviors (Riedl et al., 2015; Yang et al., 2021) or attitudes toward unaffected *observers* who intervened in face of immorality (Lee and Warneken, 2020). However, little is known about how children evaluate *victims'* responses to immorality. Learning how children evaluate victims' responses is important, as children might be the victims of immorality themselves and whether they support or oppose different responses from victims reflects their moral values and behavioral tendencies (see also Oostenbroek and Vaish, 2019, who proposed that children evaluated forgiving victims positively because they approved of repairing cooperation). Therefore, the present study aimed to examine how children evaluate victims' responses to immorality as unaffected bystanders.

Second, the punishment option in these previous studies is either very harsh (made the perpetrator lose all or most of the resources; e.g., Yang et al., 2021) or very mild and ineffective for the perpetrator (the perpetrator did not lose anything they initially owned; e.g., Riedl et al., 2015). Therefore, it remains unclear whether children genuinely prefer restoration over punishment, or they only prefer restoration when punishment is too harsh or ineffective. In fact, adults expect the degree of punishment to match the immorality of transgression, suggesting that the degree of punishment matters in adults' reasoning about justice (Wenzel and Okimoto, 2016). Specifically, if punishment is too mild to fit the transgression, it will be inefficient and unsatisfying (Adams, 2016); but if it is too severe, it will lead to other negative consequences including violence (Murphy, 2003) and further damage (McCullough et al., 2013). Prior work on how the degree of punishment corresponds to different transgressions mostly comes from a judicial perspective and only tests adult participants (Murphy, 2003; McCullough et al., 2013; Adams, 2016). Children also frequently encounter social conflicts such as unpermitted taking of toys and unequal resource distributions (Hartup et al., 1988; Laursen and Adams, 2018). However, little is known about how children weigh different degrees of punishment against restoration. Therefore, the present study aimed to systematically investigate children's relative preference between restoration and different degrees of punishment in contexts that they routinely encounter in their lives.

Studying this question with children will enrich our understanding of the origin of human justice. It will also provide insights into educational practices for the development of moral reasoning and social skills. For example, teachers and parents frequently encounter the problem of guiding children to deal with daily conflicts with peers. If children's toys are taken away by others, should teachers or parents ask children to first restore the toys back or punish the perpetrator? As children begin to develop their own views of justice and morality, understanding how they interpret and evaluate these different actions may shed light on potential solutions to this problem.

The other goal of the present study is to examine the connection between children's evaluations and their own behaviors. Previous work comparing punishment and restoration only measured children's behaviors (to punish or to restore; Riedl et al., 2015; Yang et al., 2021) or their evaluations of or attitudes toward others' behaviors (Oostenbroek and Vaish, 2019; Lee and Warneken, 2020). We do not yet know whether children's evaluations connect or disconnect with their own behaviors in the context of punishment and restoration. Theories and studies show that the knowledge–behavior gap commonly exists among adults (Rimal, 2000) and children (Blake et al., 2014; Blake, 2018) in various domains. For instance, preschool children understand fairness principles and prefer fair allocations but they do not allocate resources fairly (Rogers and Tisak, 1996; Smith et al., 2013). Given the documented gap between evaluations and behaviors (see also Kollmuss and Agyeman, 2002; Juvan and Dolnicar, 2014), we aimed to examine whether children's evaluations of punishment and restoration parallel with their actual behaviors when they are victims of transgressions.

### The Present Study

The present study investigated how children, as unaffected observers, evaluated the victims who chose punishment or restoration in the face of immorality. We tested children in possession violation cases, as children at this age are familiar with these scenarios (Hartup et al., 1988). Importantly, we included Chinese children, who were relatively less studied and may be different from the WEIRD (Western, Educated, Industrialized, Rich, and Democratic) group. Compared to WEIRD cultures, Chinese culture emphasizes more on duty-based communal obligations and spiritual purity (Buchtel et al., 2015). Therefore, different from WEIRD adults, Chinese adults think the “informal immorality controls” (which are based on moral rules rather than laws) are also important (Jiang et al., 2010). Although studies on adults find cultural differences, little is known about whether the cultural differences on justice judgement emerges in children. Studies with them could help us further understand the commonalities in children's justice development (Henrich et al., 2010). We tested children aged 4–6 because children in this age range can distinguish restoration and punishment (Riedl et al., 2015; Lee and Warneken, 2020; for studies that tested children from the same cultural background, see Yang et al., 2021).

Specifically, using a within-subject design, we presented children with scenarios in which one victim chose to restore the possessions (the restorer) and the other chose to punish the perpetrator (the punisher) in response to possession violations. Importantly, we manipulated the degree of punishment and contrasted each of them with restoration (Experiments 1–3) and among themselves (Experiment 4). We incorporated a battery of evaluation measures, including children's ratings of these different actions and their attitudes toward the victims. Testing how children evaluated others' behaviors avoids triggering potential negative emotions and self-interested tendencies (because children were not victims), thus offering a relatively neutral assessment. We hypothesized that the degrees of punishment played a role in children's relative evaluations of restoration and punishment. Children may evaluate restoration relatively more positively when punishment was too mild or too harsh (for reasons discussed above). We also further examined how different degrees of punishment compared among themselves. Additionally, in order to understand whether children's relative evaluations between punishment and restoration aligned with their own behaviors, we also asked children what they would do in a similar situation. We hypothesized that children who chose restoration themselves would especially evaluate the restorer more positively than the punisher.

## Experiment 1: Restoration vs. Harsh Retribution

### Method

#### Participants

The participants were 48 Chinese children (24 girls, *M* = 62.89 months, *SD* = 3.96, range = 56.62–72.10 months) from a private preschool in Beijing. Four additional participants were tested but excluded from data analyses for not completing the experiment. We decided this sample size based on prior work in this field (Oostenbroek and Vaish, 2019) and given our resources. *Post-hoc* power analysis using the current sample size and main results with G^*^Power 3.1 (two tails, α = 0.05, difference between two dependent means, effect size calculated from the behavior rating measure) indicated that we achieved 84% power. In all experiments reported in this paper, parents' consent forms were obtained *via* the preschool and children received a picture book for participation. This research project has Institutional Review Board approval from Tsinghua University, protocol #201602.

#### Procedure

A female experimenter tested the children individually in a quiet room. The experiment consisted of 5 phases: introduction, video watching, comprehension check, main evaluation task, followed by a behavioral task. We tested children's evaluation first because it is the main focus of the current study. If the behavioral task was completed before the evaluation, then evaluations might be changed to justify their own behavior (Bandura et al., 1996; Tsang, 2002). It took ~15 min to complete the experiment for each child. In what follows, we summarized the procedure of this experiment; exact scripts are included in Supplemental Materials (same for the following experiments).

**Figure 1:**
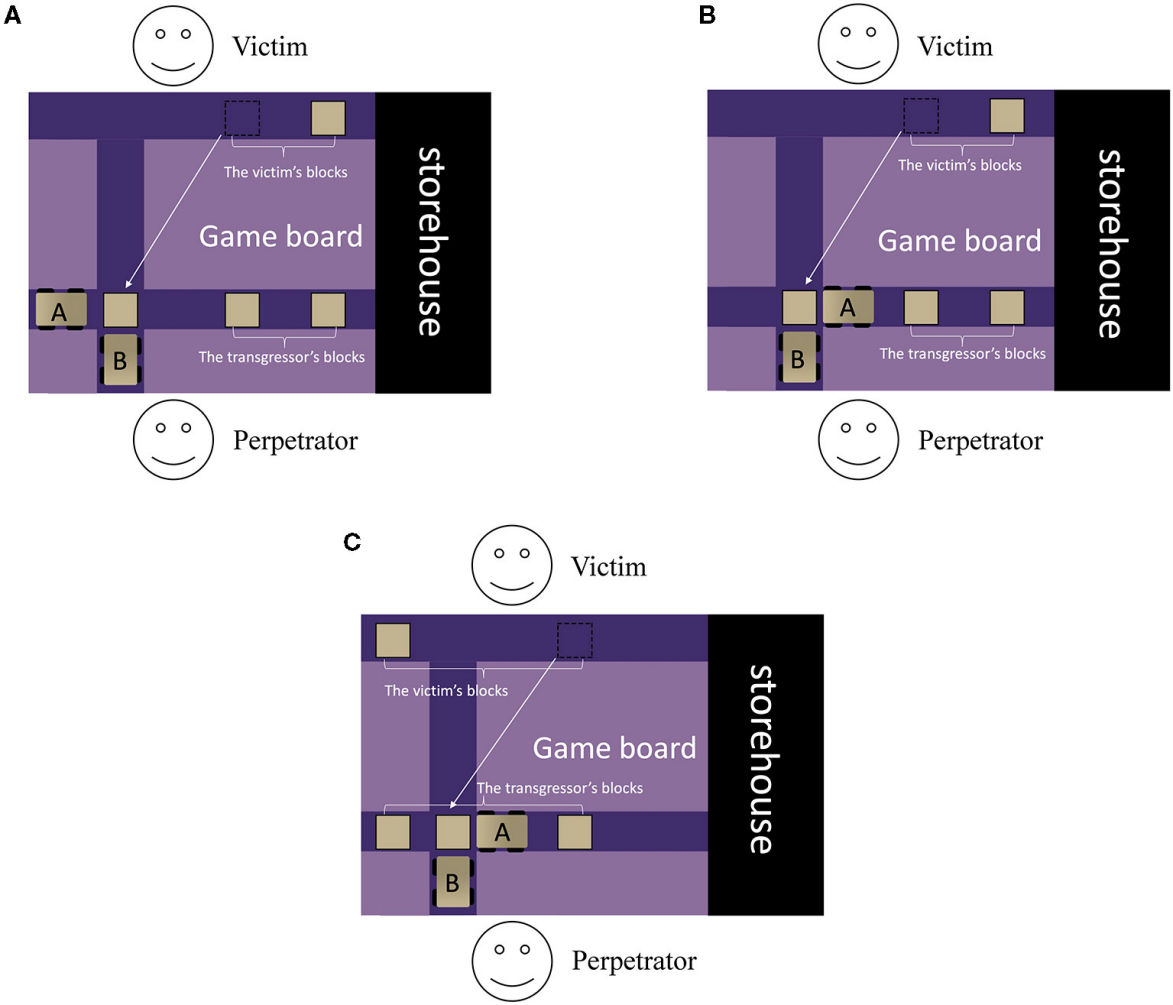
The arrangement of the game board in three experiments (see online version for the colored version of this figure). **(A–C)** represent the experimental set-up in Experiments 1, 2, and 3, respectively. The victim (top) owns the blocks (represented by white squares in the figure) on the upper horizontal lane, while the perpetrator (bottom) owns the blocks on the lower horizontal lane. There are two cars (shaded gray rectangles with four black “wheels”); these cars can move on the dark purple lanes that they are located at. Car A is on the lower horizontal lane and can move toward the “storehouse” on the right side, whereas Car B is on the vertical lane and moves upwards toward the upper horizontal lane. The white arrows illustrate the relocation of the victim's block caused by the perpetrator. The difference among the three experiments is the number of blocks that Car A can push to the storehouse. In Experiment 1 **(A)**, Car A can push 3 blocks to the storehouse, thus the perpetrator has no blocks left. In Experiment 2 **(B)**, Car A can push 2 blocks to the storehouse, thus the perpetrator has 1 block left. In Experiment 3 **(C)**, Car A can push 1 block to the storehouse, thus the perpetrator has 2 blocks left.

*Introduction*. Children were introduced to the rules of a novel game (adapted from Yang et al., 2021; see Figure 1). In this game, two players (acted by real-life puppets) faced each other across the purple game board. Each player began with two wooden blocks (toys of players) and they put their blocks on the horizontal lane closer to them. There were two cars on the game board (we added this feature to make the game more appealing to children). Children were told that each car only moved in the given direction (as demonstrated in Figure 1). Pushing different cars resulted in wooden blocks being moved to either the storehouse (blocks in the storehouse did not belong to either player, so we called this the “punishment” option), or the original location (returning the relocated blocks to where they originally belonged to, so we called this the “restoration” option). We manipulated the puppets, the game board, the cars, and the blocks in real life. To ensure that children understood the respective consequences of pushing each car, we asked them to push the cars by themselves. Children could not proceed to the next step until they answered the comprehension questions correctly (*How many blocks are left on the upper or the lower lane after moving this car?*). If they failed to correctly answer the questions, the experimenter would reintroduce the rules (*n* = 10). All children understood the rules after the experimenter reintroduced the rules once or twice.

*Video watching*. In order to standardize the procedure, each child watched both video clips that showed different events. A video sample has been uploaded to the Open Science Framework at this weblink https://osf.io/u59kd. Children were first introduced to the two real-life puppets featured in each video (learning their names and greeting them in person) before watching videos on an iPad. Each video began with two puppets facing each other as described above. Then one puppet (the victim) left for the restroom, and the other puppet (the perpetrator) took a block from the victim's lane and put it on his or her own lane. Later the victim returned, realized that his or her block was stolen by the perpetrator, and faced a decision between punishment and restoration, as specified above. In the punishment video, the victim punished the perpetrator (by moving one car); in the restoration video, the victim restored his or her block (by moving the other car). The experimenter referred to the puppets with their names (e.g., “*Hua*”) rather than “the victim,” “the perpetrator,” or “the puppet” (these words were never used with children during testing). Whether children watched the restoration video or the punishment video first was counterbalanced across children, so were the role of the puppets (acted as a perpetrator, a punisher, or a restorer).

*Comprehension Check*. After watching each video clip, the experimenter showed the real-life two puppets and the game board that appeared in the previous video clip, to help children recall the plot. Children were asked the following comprehension questions: “*Who went to the restroom?*” “*Who took a block without the other's permission?*,” “*Which car did Hua (the victim) move?*,” and “*How many blocks did Hua and Feng (the perpetrator) have at the end?*.” Those who failed to give correct answers at first try (*n* = 16 after the restoration video and *n* = 7 after the punishment video) passed the task after re-watching the video clips. More than half of the children correctly answered the comprehension questions for the first time. The times of replay in the restoration condition and the punishment condition did not differ significantly (χ^2^ (2) = 4.69, *p* = 0.10). In addition, whether children correctly answered the comprehension questions at the first time did not significantly influence children's performance in the main tasks (behavior ratings and liking scores) (*p*s > 0.05, for the detailed statistics, see Supplementary Materials).

*Main Evaluation Task*. There were three measures (in the following order): (1) behavior ratings of the four puppets. With visual aids of happy vs. unhappy faces, we asked children to evaluate each puppet's behavior (two perpetrators, and two victims—one punisher and one restorer; “*Is his or her behavior good or bad?*”) followed by a question about to what extent children considered the behavior as good or bad (“*Is it a little good, or very good?*” or “*Is it a little bad, or very bad?*”) and a justification question (“*Why do you think so?*”. For results on children's justifications, see Supplementary Materials). By measuring children's behavior ratings, we can learn whether children approve of punishment and restoration. (2) Liking scores of the two victims (the punisher and the restorer; “*Do you like him or her?*”) followed by a question about to what extent children liked or disliked the protagonist (“*Do you like him or her a little or a lot?*” or “*Do you dislike him or her a little or a lot?*”). By asking how much children like the punisher or the restorer, we can learn whether the protagonist's behavior affect children's evaluation of the protagonist. (3) Sticker allocation task between the two victims. The participant was given a sticker, and was asked which victim to give it to, the punisher or the restorer. This question was to examine whether children preferred the punisher or the restorer.

*Behavioral task*. Finally, to probe children's own behavioral responses, we asked children which option they preferred if they were victims of similar possession violations. Children were instructed to imagine playing with a classmate who took a block away when they went to the restroom (in fact, the experimenter moved the block using the above apparatus for demonstration). Children then pushed a car either to punish the classmate or to restore his or her possessions (i.e., the block).

#### Coding and Scoring

(1) Behavior ratings: there were four raw rating scores (two scores for the two perpetrators, one score for the punisher, one score for the restorer). The scores ranged from 1 to 4, with higher scores indicating more positive ratings of the behavior. (2) Liking scores: there were two raw liking scores (one for each victim), The scores ranged from 1 to 4, with higher scores indicating more positive attitudes toward the victim. For main analyses on these two measures, we further computed difference scores between the two victims (restorer-punisher; range −3 to 3); higher values indicated relatively stronger positivity toward restoration or the restorer. (3) Sticker allocation task (forced-choice between the two victims): we coded whether children gave the sticker to the punisher or the restorer, as well as counted the number of children who gave the sticker to each victim. (4) Behavioral task: we coded whether children chose punishment or restoration, as well as counted the number of children who performed each action. A second coder coded a subset of children (30% of the data, *n* = 16) on these four measures and the inter-rater reliability was perfect (κ = 1.00).

### Results

Data analyses were conducted with R version 4.0.2 (R Core Team, 2020). Preliminary analyses with linear models (gender as an independent factor and age as a covariate) showed no significant effects of gender or age on children's responses; thus, we collapsed the data across these factors. The order of video watching (i.e., punishment first or restoration first) and the times of replaying video clips did not significantly influence the results either (*p*s > 0.05; for the detailed statistics, see Supplementary Materials). Main analyses for behavior ratings and liking scores were conducted *via* linear regressions (using the package “lmerTest;” Kuznetsova et al., 2017), while for count data we used binomial or Chi-square tests (for sticker allocation and children's own behavioral task).

#### Main Evaluation Measures

On the behavior ratings, we first ensured that children distinguished the victims from the perpetrators—children rated the victims' behaviors more positively (*M* = 3.16, *SD* = 1.03) compared to the perpetrators' (*M* = 1.43, *SD* = 0.52), *B* = 1.73, *SE* = 0.11, *p* <0.001, *R*^2^ = 0.53.

**Figure 2:**
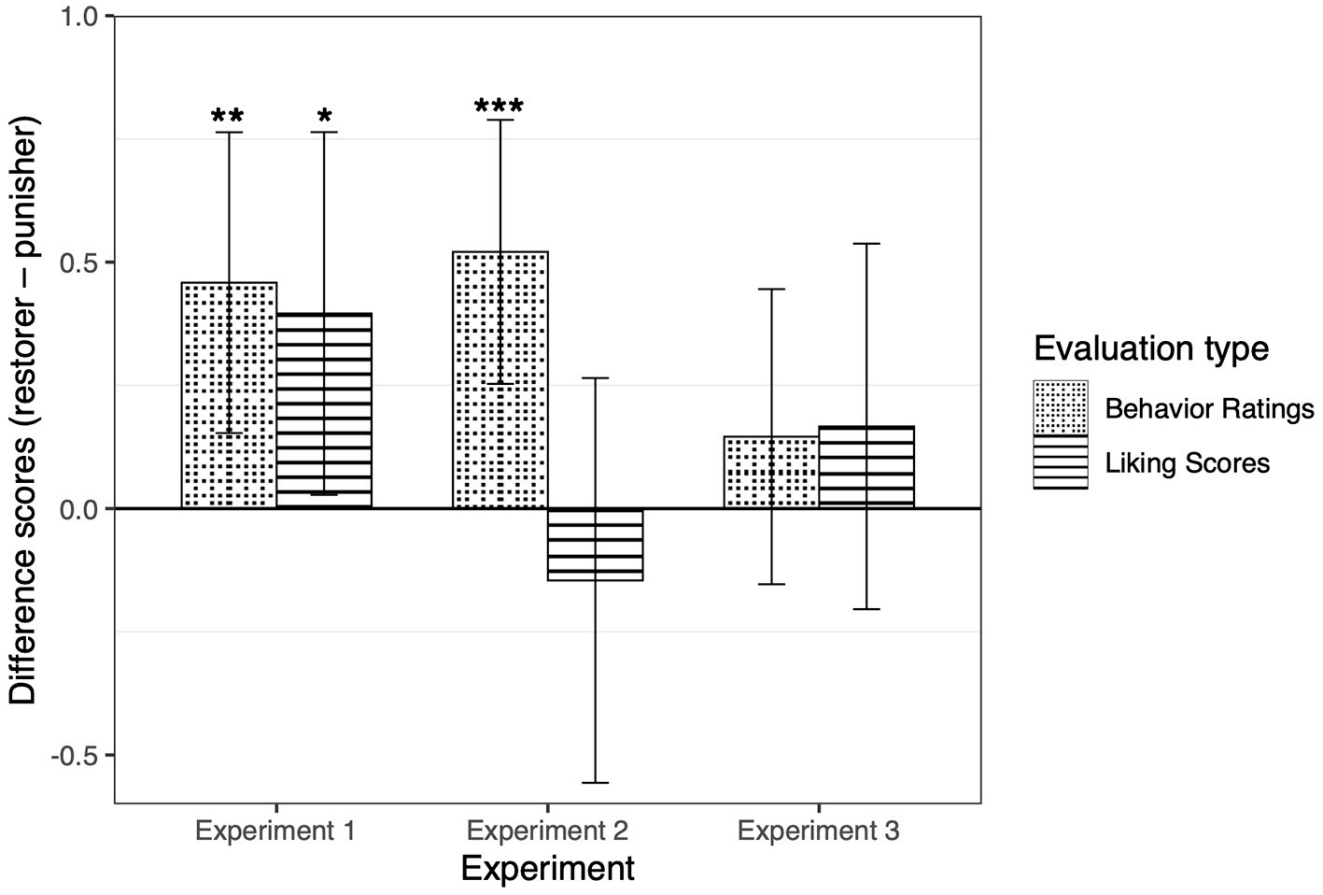
Differences of behavior ratings and liking scores between the restorer and the punisher (restorer—punisher) in Experiment 1, 2, and 3. In Experiment 1, differences of behavior ratings and liking scores were both significant, showing that children approved of the restoration behavior; in Experiment 2, only the differences of behavior ratings were significant; in Experiment 3, neither differences of behavior ratings nor liking scores was significant. The error bars represent the 95% CI. ***p <0.001, **p <0.01, * *p* <0.05.

Main analyses focused on children's relative evaluations of restoration vs. punishment (using the difference scores described above for behavior ratings and liking scores). We found that children rated restoration significantly more positively than punishment (via an intercept only model), *M*_differencescores_ = 0.46, *SD* = 1.05, *B* = 0.46, *SE* = 0.15, *p* = 0.004, Cohen's *d* = 0.44 (see Figure 2). On the liking scores (see Figure 2), children showed more favorable attitudes toward the restorer compared to the punisher, *M*_differencescores_ = 0.40, *SD* = 1.27, *B* = 0.40, *SE* = 0.18, *p* = 0.04, Cohen's *d* = 0.31 (via an intercept only model). Consistent with these results, in the sticker allocation task, children tended to give the sticker to the restorer (*n* = 31 out of 48) rather than the punisher (*n* = 17 out of 48), *p* = 0.06 (via a binomial test) (see Table 1).

**Table 1:** The number (proportion) of children who gave stickers to the punisher or the restorer in experiments 1–3.

	**Gave the sticker to the punisher**	**Gave the sticker to the restorer**
Experiment 1	17 (35%)	31 (65%)
Experiment 2	22 (46%)	26 (54%)
Experiment 3	19 (40%)	29 (60%)

*The proportion is rounded to the nearest integer*.

#### Connections Between Evaluations and Behaviors

When children's own block was taken away, 20 (out of 48, ~42%) children chose to punish the perpetrator, while 28 children chose to restore the block. A binominal test showed that the numbers of these two types of children (punitive children: children who chose punishment; restorative children: who chose restoration) were not significantly different, *p* = 0.31.

We then compared evaluation results between these two types of children for each measure (adding type of children in the previous models), as shown in Table 2. On behavior ratings, restorative children (*M*_differencescores_ = 0.75, *SD* = 0.97) rated restoration more positively than punishment (difference scores compared to 0, *p* < 0.001). Punitive children, however, did not evaluate these two behaviors differently (*M*_differencescores_ = 0.05, *SD* = 1.05, which did not differ from 0, *p* = 0.85). Restorative children also scored significantly higher on the difference scores than punitive children, *B* = −0.70, *SE* = 0.29, *p* = 0.02, *R*^2^ = 0.11. These results suggest that the relative positivity toward restoration shown above was especially driven by the restorative children.

**Table 2:** Means (standard deviations) of difference scores on behavior ratings and liking scores in experiment 1–3 as a function of children's type (punitive or restorative).

**Experiment**	**Evaluation measure**	**Children's type**	
		**Punitive children**	**Restorative children**
Experiment 1	Behavior ratings	0.05 (1.05)	0.75 (0.97)
	Liking scores	0.15 (1.09)	0.57 (1.37)
Experiment 2	Behavior ratings	0.63 (1.07)	0.45 (0.83)
	Liking scores	−0.05 (1.54)	−0.21 (1.35)
Experiment 3	Behavior ratings	0.17 (0.98)	0.14 (1.05)
	Liking scores	−0.17 (1.47)	0.21 (1.26)

On liking scores, these two types of children did not differ, *B* = −0.42, *SE* = 0.37, *p* = 0.26, *R*^2^ = 0.03 (see Table 2). They did not significantly differ in their allocations of stickers either, χ^2^ (1) = 2.19, *p* = 0.14 (see Table 3).

**Table 3:** The number (proportion) of children who gave stickers to the punisher or the restorer in experiments 1–3 as a function of children's type (punitive or restorative).

**Experiment**	**The victim that children gave the sticker to**	**Children's type**	
		**Punitive children**	**Restorative children**
Experiment 1	The punisher	10 (50%)	7 (25%)
	The restorer	10 (50%)	21 (75%)
Experiment 2	The punisher	11 (58%)	15 (52%)
	The restorer	8 (42%)	14 (48%)
Experiment 3	The punisher	5 (83%)	14 (33%)
	The restorer	1 (17%)	28 (67%)

*The proportion is rounded to the nearest integer*.

### Discussion

Experiment 1 found that children evaluated restoration more positively than punishment. Specifically, compared to punishment, children more highly rated restorative behaviors (*behavior ratings*), preferred the victim who chose restoration (*liking scores*), and tended to share the sticker with the restorer (*sticker allocations*). This finding suggests that children prioritized compensating to the victim rather than punishing the perpetrator. Interestingly, this trend was especially pronounced (at least on behavioral ratings) in children who chose restoration when facing possession violations themselves. These findings further suggest that children's behavior paralleled with their evaluation to some extent.

## Experiment 2: Restoration vs. Moderate Retribution

In Experiment 1, one alternative reason why the preschoolers viewed punishment more negatively than restoration is that removing all three of the perpetrator's blocks was too harsh. Children may have thought that even though punishment was necessary, it was inappropriate to impose such a high degree of punishment. To test this possibility, in Experiment 2, we decreased the harshness of the punishment option by changing it to removing two instead of three of the perpetrator's blocks. That is, in the punishment condition, both the perpetrator and the victim consequently got one block (see Figure 1B). The study procedure was the same as Experiment 1, except that in the ‘punishment’ video, the punisher removed 2 of the perpetrator's blocks into the storehouse.

### Participants

Another 48 Chinese children (24 girls, *M* = 64.83 months, *SD* = 3.98, range = 58.83–72.95 months) from a private kindergarten participated in this study. One additional participant was tested but not included in data analyses due to not completing the experiment.

### Coding and Scoring

Scoring and coding were the same as in Experiment 1. A second coder classified a subset (33%, *n* = 16) of the children's choices, and the inter-rater reliability was perfect, κ = 1.00.

### Results

We analyzed the data in the same way as in Experiment 1. Children again rated the perpetrators (*M* = 1.47, *SD* = 0.61) more negatively than the victims (*M* = 3.34, *SD* = 0.90), suggesting that they differentiated these behaviors, *B* = 1.88, *SE* = 0.11, *p* <0.001, *R*^2^ = 0.60.

#### Main Evaluation Measures

The difference scores on behavior ratings (*M*_differencescores_ = 0.52, *SD* = 0.92) were significantly >0 (*B* = 0.52, *SE* = 0.13, *p* < 0.001, Cohen's *d* = 0.57), suggesting that children also rated restoration more positively than punishment. As for the liking scores, however, the difference scores (*M*_differencescores_ = −0.15, *SD* = 1.41) did not significantly differ from 0 (*B* = −0.15, *SE* = 0.20, *p* = 0.48, Cohen's *d* = 0.11): children did not prefer the punisher or the restorer (see Figure 2). For the sticker allocation task, we did not find a significant difference between the numbers of children who gave the sticker to the restorer (*n* = 26) or the punisher (*n* = 22), *p* = 0.67 (see Table 1). Overall, children's preference for restoration over punishment was weaker than that in Experiment 1.

#### Connections Between Evaluations and Behaviors

When asked about what they would do as a victim, 19 (out of 48, ~40%) children chose to punish the perpetrator, while 29 children chose to restore their possessions. This difference was not significant *via* a binominal test, *p* = 0.19. Analyses comparing evaluations of restoration and punishment between these two types of children returned insignificant results (behavior ratings: *B* = 0.18, *SE* = 0.27, *p* = 0.51, *R*^2^ = 0.01; liking scores: *B* = 0.15, *SE* = 0.42, *p* = 0.71, *R*^2^ = 0.003; sticker allocation task: χ^2^ (1) = 0.02, *p* = 0.90; see Tables 2, 3). Compared to children who chose punishment in the behavioral task, those who chose restoration were not more likely to favor restoration over punishment when they evaluated victims' responses.

### Discussion

Contrasting restoration with a less harsh form of punishment, Experiment 2 again revealed that children evaluated restoration more positively than punishment when they rated these different behavioral responses to possession violations (*behavior ratings*). However, different from Experiment 1, children did not differ in their liking scores or sticker allocations between the restorer and punisher, even among children who chose restoration in the *behavioral task*. Together, these findings suggest that children's relative preference for restoration over punishment decreased as the punishment option reduced in harshness.

One alternative explanation for these differences is that the decreased level of punishment mitigated children's negativity toward the punisher. If so, when the degree of punishment is further decreased, children will evaluate punishment more similarly as restoration. Another possibility is that children actually favor one particular degree of punishment, such as a moderate degree that is not too harsh or too mild (as mild punishment could become ineffective). If so, children will show stronger negativity toward the punisher if the punishment further decreased in harshness (thus becoming ineffective). To further examine these possibilities, we conducted Experiment 3.

## Experiment 3: Restoration vs. Mild Retribution

In Experiment 3, the degree of punishment was further decreased, involving only the removal of one block from the perpetrator. That is, there was no cost to the perpetrator's original possession since the perpetrator stole one block from the victim (see Figure 1C). Therefore, after punishment, the perpetrator ended up with two blocks, while the victim only had one block left.

### Participants

We recruited another 48 Chinese children (24 girls, *M* = 61.20 months, *SD* = 4.29, range = 55.51–72.98 months) from a private kindergarten. Two additional participants were excluded from our data analyses for not completing the experiment.

### Results

We conducted the same data analyses as in the first two experiments. Again, we confirmed that children differentiated the victims (*M* = 3.09, *SD* = 0.97) from the perpetrators (*M* = 1.45, *SD* = 0.61): children's behavior ratings of both the punisher and the restorer were significantly higher than the ratings of the perpetrator in both conditions, *B* = 1.65, *SE* = 0.11, *p* <0.001, *R*^2^ = 0.51.

#### Main Evaluation Measures

The difference scores on both behavior ratings (*M*_differencescores_ = 0.15, *SD* = 1.03) and liking scores (*M*_differencescores_ = 0.17, *SD* = 1.28) did not significantly differ from 0 (behavior ratings: *B* = 0.15, *SE* = 0.15, *p* = 0.33, Cohen's *d* = 0.15; liking scores: *B* = 0.17, *SE* = 0.18, *p* = 0.37, Cohen's *d* = 0.13). In addition, although there were more children distributing the sticker to the restorer (*n* = 29) rather than to the punisher (*n* = 19), the difference was not significant, *p* = 0.19 (binomial test). Overall, children did not evaluate punishment and restoration differently.

#### Connections Between Evaluations and Behaviors

With regard to the children's own behavior as victims, 42 children chose to restore the lost block, while only 6 children chose to punish the perpetrator. This difference was significant as revealed by a binominal test (*p* < 0.001). Punitive children showed a tendency to give the sticker to the punisher (5 out of 6, or 83%) compared to restorative children (14 out of 42, or 33%, so they were more likely to give the sticker to the restorer), χ^2^ (1) = 3.60, *p* = 0.06. Their evaluations on the other two measures did not differ (*p*s > 0.50; see Table 2). However, given that very few children chose to punish the perpetrator (*n* = 6), comparisons between these two different types of children on evaluations should be interpreted more cautiously.

### Comparing Experiments 1–3

To compare children's evaluations across the three experiments, we ran a mixed linear model to predict children's difference scores as a function of experiments (1, 2, and 3) and measure types (behavior ratings and liking scores), with trials nested within children. The interactions between experiments and measure types were significant when comparing Experiment 1 with Experiment 2 (*B* = −0.60, *SE* = 0.33, *p* = 0.07, *R*^2^ = 0.10; marginally significant), and comparing Experiment 2 with Experiment 3 (*B* = 0.69, *SE* = 0.33, *p* = 0.04, *R*^2^ = 0.10). Specifically, children evaluated restoration most positively when the degree of punishment was the harshest among the three experiments (Experiment 1), because they gave significantly higher behavior ratings and liking scores to the restorer than the punisher. Children reduced this positivity when the punishment was moderate (Experiment 2), because they only gave significantly higher behavior ratings to the restorer than the punisher. When the degree of punishment was the mildest, there were no significant differences between children's evaluation (behavior ratings and liking scores) of the punisher and the restorer (Experiment 3). These results together showed that the degree of punishment influenced children's preferences between punishment and restoration—restoration was evaluated most positively when contrasting with harsh punishment, but such a priority was mitigated as the level of punishment decreased.

## Experiment 4: A Direct Comparison of Harsh, Moderate and Mild Punishment

Experiments 1 to 3 systematically examined children's relative evaluations of restoration vs. different degrees of punishment. Children preferred restoration over harsh forms of punishment, but rated them similarly when punishment was less harsh. However, as these different punishment options were always contrasted with the restoration option, it was unclear how children evaluated different degrees of punishment among themselves. Experiment 4 aimed to directly answer this question.

### Method

#### Participants

Another 40 Chinese children (17 girls, *M* = 63.63 months, *SD* = 4.61, range = 54.95–71.97 months) from a private kindergarten participated in this experiment. Two additional participants were not included in our data analyses due to not completing the experiment.

#### Mat***e***rials

The materials in Experiment 4 were similar to those in the previous experiments with three exceptions. First, we removed the toy cars that were used to move blocks in the previous experiments; instead, the experimenter moved the blocks directly by hand to reduce children's cognitive load. Second, to further make it clear why the blocks moved away from the perpetrator cannot be taken back, we replaced the name of the “storehouse” with “trash can.” Third, we only used one puppet to act as the perpetrator in all three conditions (instead of changing the perpetrator puppets across different conditions).

#### Design and Procedure

In this experiment, we demonstrated three possible actions from the punishers with three different puppets: removing one, two, or three blocks, representing mild, moderate, and harsh punishment, respectively. The procedure was similar to the previous experiments. We first introduced the rules to children, and then presented the scenarios in which the victim punished the perpetrator (e.g., removing one, two, or three blocks from the perpetrator). We used live demonstrations instead of videos because a pilot study showed that this way we can better demonstrate and contrast the three punishment actions in this experiment. At the end of each punishment story demonstration, we asked children comprehension questions: “*Who went to the restroom?*,” “*Who took a block without the other's permission?*,” “*What did Hua (the victim) do?*,” and “*How many blocks did Hua and Feng (the perpetrator) have at the end?*” If they answered incorrectly, we would repeat the story again, until they correctly answered these questions. More than 85% of children (35 children in the mild condition, 34 children in the moderate and harsh condition) answered these questions correctly at the first time.

After children answered the comprehension questions correctly, we asked for children's behavior ratings of the perpetrator and the punisher in the story that was just demonstrated. The order of the three demonstrations and the role of the puppets were counterbalanced across children. After behavior ratings, children were shown all three punishers and were asked, “*Which one is your favorite?*” They were also asked “here is a sticker. Whom do you want to give the sticker to?” The preference question and the sticker allocation task were to probe children's favorite punisher. Last, to measure children's own behavioral responses, instead of a forced choice between restoration and punishment, children were asked an open-ended question to allow for free responses (“*What will you do if someone took your block without your permission?*”).

#### Coding and Scoring

The coding and scoring for behavior ratings were similar as in the previous experiments. On children's favorite punisher and the sticker allocation task, children preferring or giving sticker to the mild punisher was coded as 1, to the moderate punisher as 2, and to the harsh punisher as 3. A second coder independently coded 33% (*n* = 13) of these data. The inter-rater reliability was perfect, κ = 1.00.

Children's behavior responses were coded into 3 categories. (1) Communication: talking to or discussing with the perpetrator; e.g., “*I will ask him to give the block back to me”* or “*I will remind her not to take my blocks without my permission”*. (2) Restoration: taking the blocks back without referencing communication; e.g., “*I will take my block back.”* (3) Punishment: retaliating against the perpetrator without referencing communication; e.g., throwing away the perpetrator's blocks or not playing with the perpetrator. Two coders classified all the responses independently, and the inter-rater reliability was good, κ = 0.96. Discrepancies were resolved through discussion.

### Results

Preliminary analyses with linear models (gender as an independent factor and age as a covariate) showed no significant effects of gender or age on children's responses; thus, we collapsed the data across these factors. The order of the three demonstrations (i.e., mild punishment first, moderate punishment first, or harsh punishment first) and the times of repeats did not significantly influence the results either (*p*s > 0.05; for the detailed statistics, see Supplementary Materials).

#### Main Evaluation Measures

**Figure 3:**
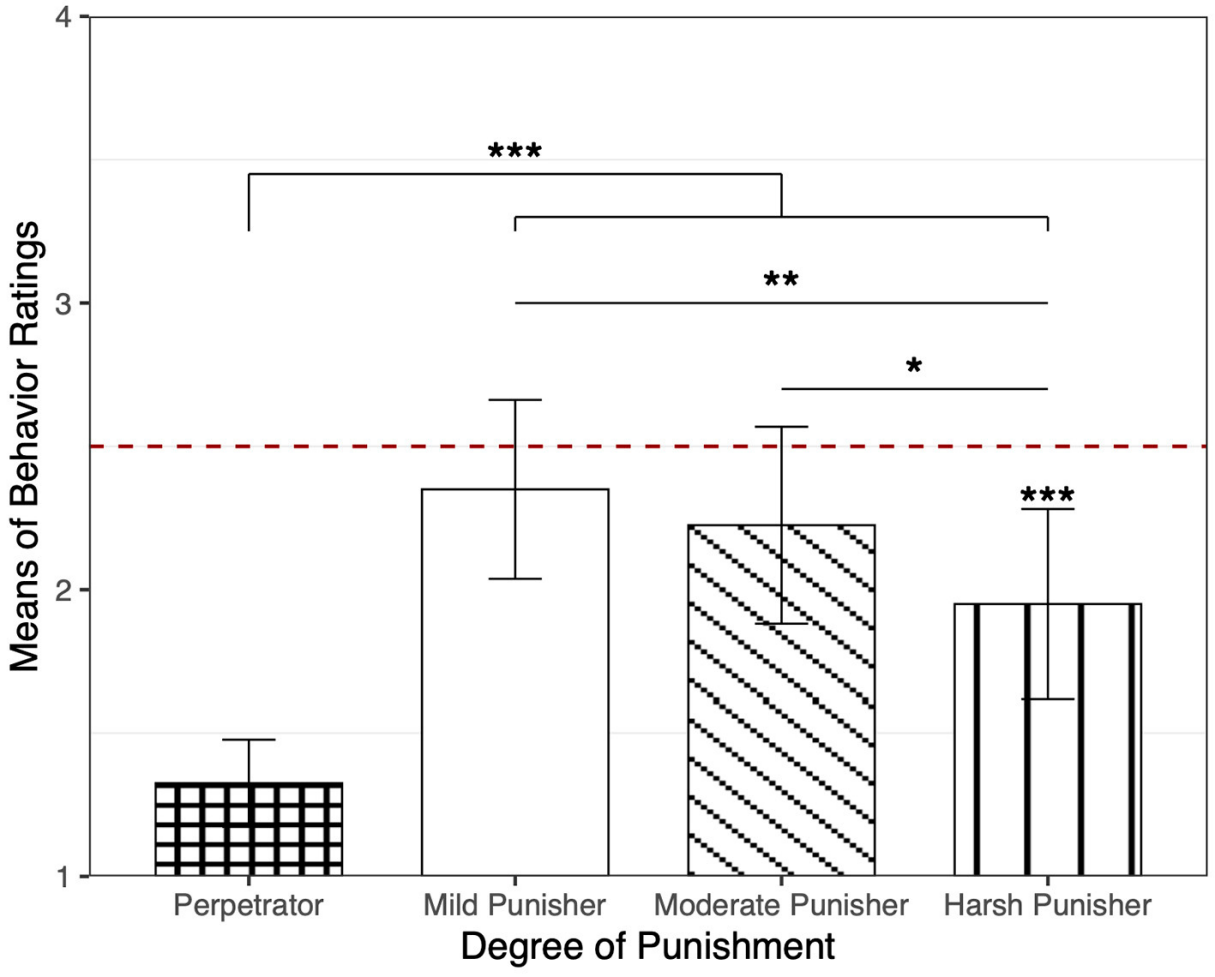
Children's behavior ratings of the 3 punishers in Experiment 4. Children's behavior ratings of all the three punishers were significantly higher than the behavior rating of the perpetrator. The behavior ratings of the mild punisher and the moderate punisher were significantly higher than behavior ratings of the harsh punisher; and the behavior rating of the harsh punisher was significantly lower than the mid-point of the rating score. Error bars represent the 95% CI. ***p < 0.001, **p < 0.01, *p < 0.05.

First, children rated the perpetrator (*M* = 1.32, *SD* = 0.47) more negatively than the three punishers (*M* = 2.17, *SD* = 1.03), *B* = 0.85, *SE* = 0.09, *p* <0.001, *R*^2^ = 0.22. Then we conducted a mixed linear model to predict children's behavior ratings of punishers (mean-centered prior to modeling) as a function of degrees of punishment (with trials nested within children). As shown in Figure 3, the effect of the degrees was significant (*p* = 0.02): children rated the harsh punisher more negatively than the mild punisher (*B* = 0.40, *SE* = 0.14, *p* = 0.006, *R*^2^ = 0.03) and the moderate punisher (*B* = 0.28, *SE* = 0.14, *p* = 0.05, *R*^2^ = 0.01), while they did not evaluate the mild and moderate punishers differently (*B* = −0.13, *SE* = 0.14, *p* = 0.38, *R*^2^ = 0.002). In fact, they rated the harsh punisher more negatively than the mid-point of the score (ranging from 1 to 4, the mid-point is 2.5), *p* = 0.001, demonstrating clear negativity toward the harsh punisher.

Results on the two forced-choice measures did not reach significance. On the question asking about children's favorite punisher, 16 (40%) children chose the mild punisher, 10 (25%) children chose the moderate punisher, and 14 (35%) children chose the harsh punisher. A Chi-square test showed that the difference was not significant, χ^2^(2) = 1.40, *p* = 0.50. As for the sticker allocation task, 12 (30%) children gave the sticker to the mild punisher, 15 (37.5%) children to the moderate punisher, and 13 (32.5%) children to the harsh punisher. A Chi-square test showed that the difference was not significant, χ^2^ (2) = 0.35, *p* = 0.84.

#### Connections Between Evaluations and Behaviors

For children's own behavior, children's responses were coded into 3 types (communication, restoration, or punishment): 17 children (42.5%) chose to communicate with the perpetrator, 4 children (10%) chose to restore the lost block, and 19 children (47.5%) chose to punish the perpetrator. Since there were only 4 children coded into the restoration type, we combined the communication type and the restoration type into the non-punitive type. The numbers of children in the punitive (*n* = 19) vs. non-punitive (*n* = 21) types were not significantly different, *p* = 0.87.

**Figure 4:**
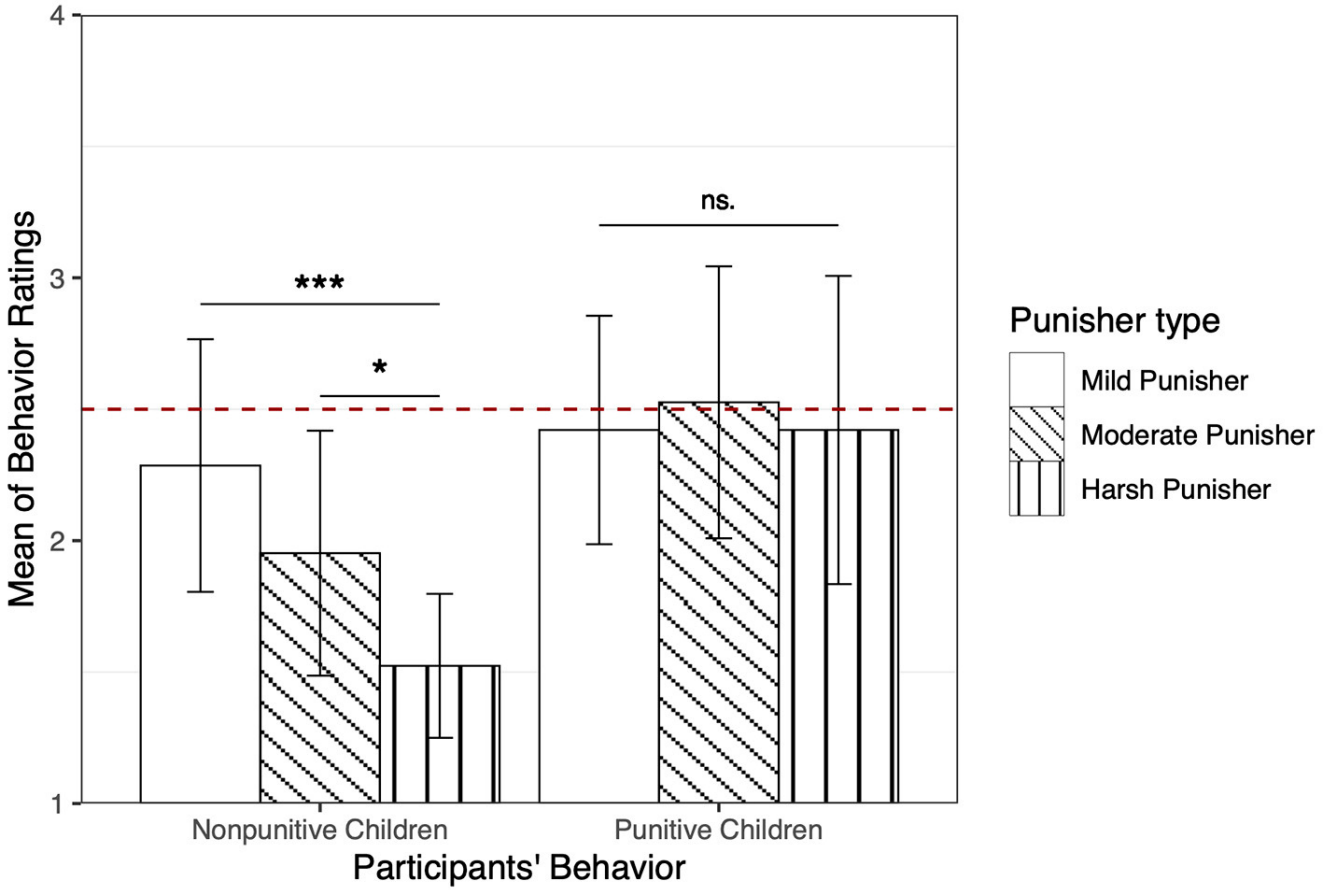
The behavior ratings of 3 punishers in two types of children whose behavior was non-punitive (i.e., communication or restoration) or punitive. For non-punitive children, their behavior ratings of the mild punisher and the moderate punisher were significantly higher than behavior ratings of the harsh punisher. For punitive children, their behavior ratings of three punishers were not significantly different. Values are expressed as the means and error bars as the 95% CI. ***p < 0.001, *p < 0.05. ns. = not significant (*p*> 0.05).

We compared the behavior ratings of the three punishers between the two major types of children (non-punitive vs. punitive) using a mixed linear model. Results revealed an interaction between the punishment degrees and children's behavior (*p* = 0.02). Simple effect analysis showed that only those children who chose not to punish the perpetrator (non-punitive type) differentially evaluated the three punishers' behaviors: they rated the harsh punisher (*M* = 1.52, *SD* = 0.60) more negatively than both the mild punisher (*M* = 2.29, *SD* = 1.06, *B* = 0.76, *SE* = 0.19, *p* <0.001, *R*^2^ = 0.05), and moderate punisher (*M* = 1.95, *SD* = 1.02, *B* = 0.43, *SE* = 0.19, *p* = 0.02, *R*^2^ = 0.02). In contrast, children classified as punitive did not rate the three punishers differently (*p*s > 0.59, see Figure 4).

We also compared results for the liking and sticker task between the two major types of children (non-punitive vs. punitive) using Chi-square tests. There was no significant difference in children's favorite punisher (χ^2^(2) = 1.79, *p* = 0.42), nor was there difference on the sticker task (χ^2^(2) = 0.38, *p* = 0.83), between the two types of children (see Table 4). Overall, on these two forced-choice measures punitive and non-punitive children did not respond differently.

**Table 4:** The number (proportion) of two types of children's favorite punisher and of children who gave stickers to each punisher in experiment 4.

**Evaluation measure**	**Punisher**	**Children's type**	
		**Punitive children**	**Non-punitive children**
Children's favorite punisher	Mild punisher	8 (42%)	8 (38%)
	Moderate punisher	3 (16%)	7 (33%)
	Harsh punisher	8 (42%)	6 (29%)
The punisher that children gave the sticker to	Mild punisher	5 (26%)	7 (33%)
	Moderate punisher	7 (37%)	8 (38%)
	Harsh punisher	7 (37%)	6 (29%)

*The proportion is rounded to the nearest integer*.

### Discussion

Consistent with the Experiment 1, 2, and 3, here when children directly compared different degrees of punishment among themselves, they evaluated the milder degrees of punishment more positively than the harsh one (on behavior ratings). This result was driven by children who chose not to punish the perpetrator themselves in a free-response behavior task. The other two measures (preference and sticker allocation) returned largely insignificant results. It is possible that although children rated the harsh punishment behavior as wrong, they did not necessarily dislike the harsh punisher or refrain from rewarding the harsh punisher with stickers. For example, children may not agree with retaliation behavior, but they like to play with these “retaliators” (Etchu, 2005). Another possibility is that forced-choice measures and non-parametric tests (on liking and sticker giving) were less sensitive compared to continuous ratings and parametric tests (on behavioral rating). Future studies are needed to further investigate these possibilities.

## General Discussion

There has been a hot debate on when (under what condition) and how people uphold justice (Heffner and FeldmanHall, 2019). The current study approached these issues from a developmental perspective, offering insights into the basic human tendencies in punishment and restoration. There are three main findings. First, extending prior work (Riedl et al., 2015; Yang et al., 2021), we demonstrated that 4–6-year-old children evaluated restoration more positively than punishment when facing possession violations, but only when punishment was relatively harsh. We are the first to show that with the decrease in the degree of punishment, children reduced their relative preference for restoration over punishment (Experiments 1–3). These new findings thus necessitate the need to consider multiple factors (such as the degree of severity) when studying children's evaluations of restoration and punishment. Second, when directly comparing different degrees of punishment (Experiment 4), children also evaluated the milder punishment options more positively than the harsh one. These findings again show that the degree of punishment matters in children's evaluations, and milder forms of punishment may be preferred by children. Third, across experiments the positive evaluations of restoration were especially driven by children who chose to restore when their possessions were violated, suggesting links between evaluations and behaviors in the context of justice restoration.

The finding that children overall preferred restoration over punishment is consistent with previous work that children preferred restoration rather than punishment (Riedl et al., 2015; Lee and Warneken, 2020; Yang et al., 2021). Note that previous studies found that children prefer restoration than punishment in different contexts (Riedl et al., 2015; Lee and Warneken, 2020; Yang et al., 2021). Here we further show that children preferred restoration conditionally, only when punishment was relatively harsh. Specifically, when punishment was harsh (removing 3 blocks when the perpetrator stole 1 block), children evaluated restoration more positively than punishment across three evaluation measures. In contrast, when punishment was moderate or mild (i.e., removing 2 or 1 blocks), children evaluated punishment more similarly as restoration. However, different measures (i.e., behavior ratings, liking scores, and sticker allocation) yielded different results. It may be because the behavior ratings examined evaluation of the punishment and restoration behaviors, while the liking scores and sticker allocations examined whether children liked the punisher or the restorer. Studies have shown that children aged 4–5 can understand that the traits of a person is stable over time (Liu et al., 2007), and the one-time behavior may not affect children's liking of the protagonist. For example, although children judged the retaliation behavior as wrong, they would like to play with the one who retaliated the perpetrator as a victim, suggesting a dissociation between behavioral evaluation and liking (Etchu, 2005). Consistently, the present study revealed children's different attitudes toward punishment and restoration behaviors, while they did not necessarily vary their liking for the protagonists, potentially because the latter might be relatively stable and did not easily change due to a one-time action.

In addition, when directly contrasting these three different degrees of punishment, children negatively evaluated harsh punishment (compared to the mid-point of the scores), more so than the other two milder forms of punishment, perhaps because harsh punishment did not correspond to the severity of the perpetration. It further showed an urge for retributive justice to ensure that offenders receive a fair degree of punishment (Wenzel et al., 2008). Also, the results showed that the degree of punishment matters in children's evaluation, which is consistent with adults (Murphy, 2003; McCullough et al., 2013; Adams, 2016; Wenzel and Okimoto, 2016).

Taken together, the current study is one of the first to suggest that preschoolers are sensitive to the severity of punishment, and they evaluate different degrees of punishment differently, perhaps in light of the norm violation (in our case, since the violation is relatively minor, they refrain from harsh punishment). Although punishment is effective in increasing compliance with norms (Fehr and Gächter, 2002), it is critical to impose punishment fairly. These results also correspond to previous studies that find that children prefer rational punishment (punishment that brought a fair outcome) than the irrational punishment (punishment that brought an unfair outcome) (Lee and Warneken, 2020). Taken together, children as young as age 4 are prudent in sanctioning others, especially showing negativity toward harsh punishment when perpetration is relatively mild. These findings enrich our understanding of the early development of punishment and restoration, and the critical role of the severity of punishment in children's reasoning about justice restoration.

Importantly, the current study further explored the relationship between children's evaluations and their own behaviors when they faced the possession violations themselves. Overall, children who chose restoration when they were victims also evaluated restoration more positively than punishment, whereas children who chose punishment did not evaluate these two differently (this was especially pronounced in Experiment 1). When the three different degrees of punishment were directly compared (as in Experiment 4), children who chose restoration evaluated milder forms of punishment more positively than harsh punishment, while children who chose punishment did not show this pattern. These findings suggest that children's evaluations and behaviors were largely consistent (but we note that we did not always find this relationship in every experiment). The consistency suggests that children who chose to restore items rather than punishing the perpetrator prioritized the value of compensating the victim, while children who chose to punish the perpetrator recognized both the values of compensation and punishment. It is possible that children who prioritize the value of compensation or punishment have different levels of cognitive and social skills, such as self-regulation (Blake, 2018), empathic concerns (Leliveld et al., 2008), and moral reasoning (Rholes and Bailey, 1983), which account for their different degrees of gap between evaluation and behavior. For instance, children with more advanced self-regulation skills show a higher consistency between evaluation and sharing behavior responses (Blake, 2018). This possibility warrants future investigation. Another possible explanation is that in our study, the harsh punishment option removed all three blocks from the perpetrator, so it might be seen as a particularly harming action. Children generally judge harming actions as bad (Rule and Duker, 1973; Leon, 1982), and those who chose restoration might see them as strongly negative. However, children who chose punishment might be high on trait aggression (Eriksson et al., 2016), and so they might not negatively evaluate harming actions (Huesmann and Guerra, 1997; Vernberg et al., 1999). By contrast, the restoration option only involved taking one block from the perpetrator and returning it to the victim, thus it helped the victim while not strongly harming the perpetrator. Children generally judge helping behaviors as good (Van de Vondervoort and Hamlin, 2017; Lee and Warneken, 2020), and even children who chose punishment did not hold any more negativity toward restoration. Such an explanation also fits with the finding that children who chose restoration did not differentially evaluate mild punishment and restoration, since mild punishment (removing one block) was not particularly harmful for the perpetrator.

Interestingly, when comparing restoration with mild punishment (i.e., removing 1 block from the perpetrator in Experiment 3), children did not differentially evaluate restoration and mild punishment. However, very few children chose mild punishment (*n* = 6, a total number was 48) when they themselves faced possession violations (consistent with Riedl et al., 2015). One possible explanation is that preschool children have a self-interested desire to maximize their own gain (Blake and McAuliffe, 2011; Zhao et al., 2019), which drive restoration behaviors when their own interest was at stake in the behavior task; however, this desire did not influence third-party evaluations in the same manner (Blake et al., 2014). Another possible explanation of this discrepancy is that children would like to have more resources than others when they are the recipient or participant of the resource allocation (Smith et al., 2013; Sheskin et al., 2014). As mild punishment in our design resulted in children having fewer resources than the perpetrator, children avoided being at a relative disadvantage by choosing restoration in the behavioral task. A third possible explanation for the lack of differences on evaluations is that neither options here directly harm the perpetrator's possession, which may be seen as similarly generous or forgiving and thus are both beneficial for later cooperation (Oostenbroek and Vaish, 2019).

Overall, the above results were based on Chinese children, a non-WEIRD sample. These findings were in line with, and further extended conceptual scope of previous studies on WEIRD children using different methods (e.g., Riedl et al., 2015; Lee and Warneken, 2020). This consistency suggests that the early take on the preferred way to uphold justice—preferring restoration over punishment—may be similar across cultures. One possible explanation of cross-cultural similarity is that this sense of restorative justice originates from human's desire for cooperation (Rand et al., 2009), which might be cultural universal (Fehr and Schmidt, 1999). Nevertheless, the present study did not directly compare the performance of Chinese children and WEIRD children using the same methodologies. Therefore, the hypothesis of ‘cultural universalism of children’s justice understanding' should be considered prudently and it warrants future investigation. In addition, social factors including children's demographic background may influence children's preferences, as both ours and the existing studies on this issue were conducted in children that came from middle-class families. For example, studies have shown that children with high socioeconomic status are more likely to perform altruistically (Benenson et al., 2007), which may lead them to compensate the victims first. Higher maternal education level predicts children's better moral reasoning (Hinnant et al., 2013), which may lead children to consider the degree of punishment seriously. The relationship between children's sense of justice and their social background can be further studied.

While opening up interesting avenues for future research, there are several limitations in the present study. First, when examining children's behaviors, we asked them to imagine the situation (i.e., a classmate took away their block) and the experimenter did a pretend play. This is a commonly used paradigm in developmental studies (Dodge, 1980; Ball et al., 2017) that provides valuable information about children's behavioral tendencies, but children did not interact with their real classmates. Future work could explore how children behave or evaluate these different actions differently when they experience real-world perpetrations. Would the harsh punisher in the real life be less welcomed (e.g., children do not like play with them) in school? Investigating these issues will inform potential ways to teach children how to solve peer conflicts given that taking away others' toys is one of the most frequent conflicts that occur among preschoolers (Hartup et al., 1988; Laursen and Adams, 2018). Second, in our work the perpetration was a relatively minor form of possession violations (stealing or unpermitted taking of one non-valuable object). Perhaps it was not considered as severe so that children did not prefer punishing the perpetrator. This minor form of immorality is frequently seen in children's social life but perhaps not considered as severe. Future research could include more severe events (e.g., stealing a valuable object or a large quantity of objects) and perpetration in other domains (outside of possession violations) to explore children's evaluations of punishment and restoration as well as their behaviors across settings and domains. Third, the degrees of punishment (mild, moderate, and harsh) in the present study were defined by removing 1, 2 or 3 block(s) from the perpetrator. However, we did not directly ask children whether they thought the degrees were mild or harsh. Future research could benefit from examining children's judgments of severity and appropriateness of different punishment options. Lastly, as the restoration option in the present study brought a fair outcome for the victims and perpetrator, children might prefer restoration over punishment due to inequity aversion (Blake et al., 2014). However, in Experiment 2, the punishment option also resulted in an equal outcome but children still rated restoration more positively than punishment. This finding suggests that inequity aversion may not be the main factor accounting for our results. Nevertheless, future research could examine whether children still prefer restoration even when it elicits an unfair outcome.

In sum, the current study examined how children evaluated punishment and restoration behaviors as well as the victims who performed these behaviors in the face of possession violations. As observers, children overall evaluated restoration (and the restorer) more positively than punishment (and the punisher), especially when punishment was relatively harsh. Also, children rated mild and moderate punishment more positively than the harsh one, directly demonstrating the influence of the degrees of punishment on children's evaluations. This tendency to prefer restoration and milder forms of punishment was especially pronounced in children who choose to restore when they were victims, suggesting consistencies between evaluations and behaviors. By studying preschoolers' evaluations of punishment and restoration and their own behaviors when facing possession violations, this study advances our understanding of the early development of restorative and retributive justice. The findings that harsh punishment is seen as worse than restoration and milder punishment also have implications for moral education. Our research also provides valuable psychological insights into the growing interest in victim compensation as an alternative to perpetrator punishment.

## Data Availability Statement

The datasets presented in this study and the Supplementary Materials can be found in online repositories. The names of the repository/repositories and accession number(s) can be found below: https://osf.io/u59kd.

## Ethics Statement

The studies involving human participants were reviewed and approved by Tsinghua IRB. Written informed consent to participate in this study was provided by the participants' legal guardian/next of kin.

## Author Contributions

ZW conceived of the presented idea and supervised the project. ZW, XY, and XL designed the experiments and created the materials. XL performed the experiments. XL and XY analyzed the data. All the authors wrote the manuscript and approved the submitted version.

## Conflict of Interest

The authors declare that the research was conducted in the absence of any commercial or financial relationships that could be construed as a potential conflict of interest.

## Publisher's Note

All claims expressed in this article are solely those of the authors and do not necessarily represent those of their affiliated organizations, or those of the publisher, the editors and the reviewers. Any product that may be evaluated in this article, or claim that may be made by its manufacturer, is not guaranteed or endorsed by the publisher.

## References

[B1] Adams G. S. (2016). Asymmetries between victims' and transgressors' perspectives following interpersonal transgressions. Soc. Pers. Psychol. Comp. 10, 722–735. 10.1111/spc3.12291

[B2] Ball C. L. Smetana J. G. Sturge-Apple M. L. (2017). Following my head and my heart: integrating preschoolers' empathy, theory of mind, and moral judgments. Child Dev. 88, 597–611. 10.1111/cdev.12605 27557797

[B3] Bandura A. Barbaranelli C. Caprara G. V. Pastorelli C. (1996). Mechanisms of moral disengagement in the exercise of moral agency. J. Pers. Soc. Psychol. 71, 364–374. 10.1037/0022-3514.71.2.364

[B4] Benenson J. F. Pascoe J. Radmore N. (2007). Children's altruistic behavior in the dictator game. Evol. Hum. Behav. 28, 168–175. 10.1016/j.evolhumbehav.2006.10.00324265820

[B5] Blake P. R. (2018). Giving what one should: explanations for the knowledge-behavior gap for altruistic giving. Curr. Opin. Psychol. 20, 1–5. 10.1016/j.copsyc.2017.07.041 28822896

[B6] Blake P. R. McAuliffe K. (2011). “I had so much it didn't seem fair”: eight-year-olds reject two forms of inequity. Cognition 120, 215–224. 10.1016/j.cognition.2011.04.006 21616483

[B7] Blake P. R. McAuliffe K. Warneken F. (2014). The developmental origins of fairness: the knowledge–behavior gap. Trends Cogn. Sci. 18, 559–561. 10.1016/j.tics.2014.08.003 25175834

[B8] Boyles D. Carusi T. Attick D. (2008). Historical and critical interpretations of social justice, in Handbook of Social Justice in Education. Routledge.

[B9] Buchtel E. E. Guan Y. Peng Q. Su Y. Sang B. Chen S. X.. (2015). Immorality east and west: are immoral behaviors especially harmful, or especially uncivilized? Pers. Soc. Psychol. Bull. 41, 1382–1394. 10.1177/0146167215595606 26253486

[B10] Capraro V. Perc M. (2021). Mathematical foundations of moral preferences. J. Roy. Soc. Interface 18:20200880. 10.1098/rsif.2020.0880 33561377 PMC8086879

[B11] Clutton-Brock T. H. Parker G. A. (1995). Punishment in animal societies. Nature 373, 209–216. 10.1038/373209a0 7816134

[B12] Cohen R. L. (1991). Membership, intergroup relations, and justice, in Social Justice in Human Relations, Vol 1: Societal and Psychological Origins of Justice, eds Vermunt R. Steensma H. (New York, NY: Plenum Press), 239–258.

[B13] Dodge K. A. (1980). Social cognition and children's aggressive behavior. Child Dev. 51, 162–170. 10.2307/1129603 7363732

[B14] Eriksson K. Andersson P. A. Strimling P. (2016). Moderators of the disapproval of peer punishment. Group Process. Intergr. Relat. 19, 152–168. 10.1177/1368430215583519

[B15] Etchu K. (2005). Preschoolers' moral judgments about provocative, retaliative, and punitive aggression in hypothetical situations. Jap. J. Educ. Psychol. 53, 479–490. 10.5926/jjep1953.53.4_479

[B16] Fehr E. Gächter S. (2002). Altruistic punishment in humans. Nature 415, 137–140. 10.1038/415137a 11805825

[B17] Fehr E. Schmidt K. M. (1999). A theory of fairness, competition, and cooperation. Q. J. Econ. 114, 817–868. 10.1162/003355399556151

[B18] FeldmanHall O. Sokol-Hessner P. Bavel J. J. V. Phelps E. A. (2014). Fairness violations elicit greater punishment on behalf of another than for oneself. Nat. Commun. 5:6306. 10.1038/ncomms6306 25350814 PMC4266485

[B19] Fowler J. H. (2005). Altruistic punishment and the origin of cooperation. Proc. Nat. Acad. Sci. 102, 7047–7049. 10.1073/pnas.0500938102 15857950 PMC1100778

[B20] Hamlin J. K. Wynn K. Bloom P. Mahajan N. (2011). How infants and toddlers react to antisocial others. Proc. Natl. Acad. Sci. USA. 108, 19931–19936. 10.1073/pnas.1110306108 22123953 PMC3250174

[B21] Hartup W. W. Laursen B. Stewart M. I. Eastenson A. (1988). Conflict and the friendship relations of young children. Child Dev. 59:1590. 10.2307/1130673 3208570

[B22] Heffner J. FeldmanHall O. (2019). Why we don't always punish: preferences for non-punitive responses to moral violations. Sci. Rep. 9, 1–13. 10.1038/s41598-019-49680-2 31519991 PMC6744396

[B23] Henrich J. Heine S. J. Norenzayan A. (2010). The weirdest people in the world? Behav. Brain Sci. 33, 61–83. 10.1017/S0140525X0999152X 20550733

[B24] Henrich J. McElreath R. Barr A. Ensminger J. Barrett C. Bolyanatz A.. (2006). Costly punishment across human societies. Science 312, 1767–1770. 10.1126/science.1127333 16794075

[B25] Herrmann B. Thöni C. Gächter S. (2008). Antisocial punishment across societies. Science 319, 1362–1367. 10.1126/science.1153808 18323447

[B26] Hinnant J. B. Nelson J. A. O'Brien M. Keane S. P. Calkins S. D. (2013). The interactive roles of parenting, emotion regulation and executive functioning in moral reasoning during middle childhood. Cogn. Emot. 27, 1460–1468. 10.1080/02699931.2013.789792 23650955 PMC3751970

[B27] Hofmann W. Brandt M. J. Wisneski D. C. Rockenbach B. Skitka L. J. (2018). Moral punishment in everyday life. Pers. Soc. Psychol. Bull. 345, 1340–1343. 10.1177/0146167218775075 29848212

[B28] Huesmann L. R. Guerra N. G. (1997). Children's normative beliefs about aggression and aggressive behavior. J. Pers. Soc. Psychol. 72, 408–419. 10.1037/0022-3514.72.2.408 9107008

[B29] Jiang S. Lambert E. Jenkins M. (2010). East meets west: Chinese and U.S. college students' views on formal and informal crime control. Int. J. Offend. Therapy Comp. Criminol. 54, 264–284. 10.1177/0306624X08330191 19171693

[B30] Jordan J. J. Hoffman M. Bloom P. Rand D. G. (2016). Third-party punishment as a costly signal of trustworthiness. Nature 530, 473–476. 10.1038/nature16981 26911783

[B31] Juvan E. Dolnicar S. (2014). The attitude-behaviour gap in sustainable tourism. Annal. Tour. Res. 48, 76–95. 10.1016/j.annals.2014.05.012

[B32] Kanakogi Y. Inoue Y. Matsuda G. Butler D. Hiraki K. Myowa-Yamakoshi M. (2017). Preverbal infants affirm third-party interventions that protect victims from aggressors. Nat. Hum. Behav. 1, 1–7. 10.1038/s41562-016-0037

[B33] Kenward B. Osth T. (2015). Five-year-olds punish antisocial adults. Aggress. Behav. 41, 413–420. 10.1002/ab.21568 26918430

[B34] Kollmuss A. Agyeman J. (2002). *Mind* the Gap: Why do people act environmentally and what are the barriers to pro-environmental behavior? Environ. Educ. Res. 8:239–260. 10.1080/13504620220145401

[B35] Krasnow M. M. Delton A. W. Cosmides L. Tooby J. (2016). Looking under the hood of third-party punishment reveals design for personal benefit. Psychol. Sci. 27, 405–418. 10.1177/0956797615624469 26851057

[B36] Kurzban R. DeScioli P. O'Brien E. (2007). Audience effects on moralistic punishment. Evol. Hum. Behav. 28, 75–84. 10.1016/j.evolhumbehav.2006.06.001

[B37] Kuznetsova A. Brockhoff P. Christensen R. (2017). lmerTest Package: tests in linear mixed effects models. J. Stat. Softw. 27. 10.18637/jss.v082.i13

[B38] Laursen B. Adams R. (2018). Conflict between peers, in Handbook of peer interactions, relationships, and groups (2nd ed.), eds Bukowski W. M. Laursen B. Rubin K. H. (New York, NY: Guilford Press), 265–283.

[B39] Lee Y. Warneken F. (2020). Children's evaluations of third-party responses to unfairness: children prefer helping over punishment. Cognition 205:104374. 10.1016/j.cognition.2020.104374 32819708

[B40] Leliveld M. C. van Dijk E. van Beest I. (2008). Altruistic compensation vs. altruistic punishment: how people restore justice. SSRN Electron. J. 1298. 10.2139/ssrn.1298584

[B41] Leon M. (1982). Rules in children's moral judgments: Integration of intent, damage, and rationale information. Develop. Psychol. 18, 835–842. 10.1037/0012-1649.18.6.835

[B42] Liu D. Gelman S. A. Wellman H. M. (2007). Components of young children's trait understanding: Behavior-to-trait inferences and trait-to-behavior predictions. Child Develop. 78, 1543–1558. 10.1111/j.1467-8624.2007.01082.x 17883447

[B43] Lotz S. Okimoto T. G. Schlösser T. Fetchenhauer D. (2011). Punitive versus compensatory reactions to injustice: emotional antecedents to third-party interventions. J. Exp. Soc. Psychol. 47, 477–480. 10.1016/j.jesp.2010.10.004

[B44] McAuliffe K. Jordan J. J. Warneken F. (2015). Costly third-party punishment in young children. Cognition 134(Supplement C), 1–10. 10.1016/j.cognition.2014.08.013 25460374

[B45] McCullough M. E. (2008). Beyond Revenge: The Evolution of the Forgiveness Instinct. San Francisco, CA: Jossey-Bass.

[B46] McCullough M. E. Kurzban R. Tabak B. A. (2013). Cognitive systems for revenge and forgiveness. Behav. Brain Sci. 36:1. 10.1017/S0140525X11002160 23211191

[B47] Murphy J. G. (2003). Getting Even: Forgiveness and its Limits. New York, NY: Oxford University Press.

[B48] Oostenbroek J. Vaish A. (2019). The benefits of forgiving: young children respond positively to those who forgive. J. Exp. Psychol: Gen. 148, 1914–1924. 10.1037/xge0000576 31021151

[B49] Perc M. (2016). Phase transitions in models of human cooperation. Phy. Lett. A 380, 2803–2808. 10.1016/j.physleta.2016.06.017

[B50] Peterson C. Seligman M. E. P. (2004). Character Strengths and Virtues: A Handbook and Classification (1st edition). New York, NY: Oxford University Press,.

[B51] R Core Team (2020). R: A Language and Environment for Statistical Computing. R Foundation for Statistical Computing. Available online at: https://www.R-project.org/

[B52] Rand D. G. Dreber A. Ellingsen T. Fudenberg D. Nowak M. A. (2009). Positive interactions promote public cooperation. Science 325, 1272–1275. 10.1126/science.1177418 19729661 PMC2875121

[B53] Rholes W. S. Bailey S. (1983). The effects of level of moral reasoning on consistency between moral attitudes and related behaviors. Soc. Cogn. 2, 32–48. 10.1521/soco.1983.2.1.32

[B54] Riedl K. Jensen K. Call J. Tomasello M. (2015). Restorative justice in children. Curr. Biol. 25, 1731–1735. 10.1016/j.cub.2015.05.014 26096976

[B55] Rimal R. N. (2000). Closing the knowledge–behavior gap in health promotion: the mediating role of self-efficacy. Health Commun. 12, 219–237. 10.1207/S15327027HC1203_01 10938914

[B56] Rogers M. J. Tisak M. S. (1996). Children's reasoning about responses to peer aggression: victim's and witness's expected and prescribed behaviors. Aggress. Behav. 22, 259–269. 10.1002/(SICI)1098-2337(1996)22:4<259::AID-AB2>3.0.CO;2-G

[B57] Rule B. G. Duker P. (1973). Effects of intentions and consequences on children's evaluations of aggressors. J. Person. Soc. Psychol. 27, 184–189. 10.1037/h0034771 4723966

[B58] Salali G. D. Juda M. Henrich J. (2015). Transmission and development of costly punishment in children. Evol. Hum. Behav. 36, 86–94. 10.1016/j.evolhumbehav.2014.09.004

[B59] Sheskin M. Bloom P. Wynn K. (2014). Anti-equality: social comparison in young children. Cognition 130, 152–156. 10.1016/j.cognition.2013.10.008 24291266 PMC3880565

[B60] Smith C. E. Blake P. R. Harris P. L. (2013). I should but I won't: Why young children endorse norms of fair sharing but do not follow them. PLoS ONE 8:e59510. 10.1371/journal.pone.0059510 23527210 PMC3603928

[B61] Tsang J.-A. (2002). Moral rationalization and the integration of situational factors and psychological processes in immoral behavior. Rev. Gen. Psychol. 6, 25–50. 10.1037/1089-2680.6.1.25

[B62] Tyler T. R. (2009). Legitimacy and rule adherence: a psychological perspective on the antecedents and consequences of legitimacy, in The Psychology of Justice and Legitimacy (New York, NY: Psychology Press).

[B63] Vaish A. Missana M. Tomasello M. (2011). Three-year-old children intervene in third-party moral transgressions. Br. J. Develop. Psychol. 29, 124–130. 10.1348/026151010X532888 21288257

[B64] Van de Vondervoort J. W. Hamlin J. K. (2017). Preschoolers' social and moral judgments of third-party helpers and hinderers align with infants' social evaluations. J. Exp. Child Psychol. 164, 136–151. 10.1016/j.jecp.2017.07.004 28822295

[B65] Vernberg E. M. Jacobs A. K. Hershberger S. L. (1999). Peer victimization and attitudes about violence during early adolescence. J. Clin. Child Psychol. 28, 386–395. 10.1207/S15374424jccp280311 10446688

[B66] Wenzel M. Okimoto T. G. (2016). Retributive justice, in Handbook of Social Justice Theory and Research, eds Sabbagh C. Schmitt M. (New York, NY: Springer), 237–256.

[B67] Wenzel M. Okimoto T. G. Feather N. T. Platow M. J. (2008). Retributive and restorative justice. Law Hum. Behav. 32, 375–389. 10.1007/s10979-007-9116-6 17957457

[B68] Wu Z. Gao X. (2018). Preschoolers' group bias in punishing selfishness in the Ultimatum Game. J. Exp. Child Psychol. 166, 280–292. 10.1016/j.jecp.2017.08.015 28961488

[B69] Yang X. Wu Z. Dunham Y. (2021). Children's restorative justice in an intergroup context. Soc. Develop. 12508. 10.1111/sode.12508

[B70] Zhao L. Sun W. Jia X. He X. Liu Y. Lee K.. (2019). Young children selectively ignore quality to promote self-interest. J. Exp. Child Psychol. 188:104679. 10.1016/j.jecp.2019.104679 31499456

