# Supplementary material for: To Punish or to Restore: How Children Evaluate Victims' Responses to Immorality
Source: Front Psychol. 2021 Aug 13;12:696160. doi: 10.3389/fpsyg.2021.696160 (PMC8414137; doi:10.3389/fpsyg.2021.696160)
Supplement: Supplementary file 1 [file Data_Sheet_1.pdf]

Supplementary Material

1 Scripts used in Experiment 1, 2, and 3 (Chinese translations)

1.1 Introduction

> "Hello, <child's name>. Welcome! I will tell you 2 stories about some children playing games. Now let me first introduce the game rules to you. Look, this is a game board with four wooden blocks on it."

The experimenter pointed at the upper lane and said "*the child on this side has two wooden blocks, and he or she put them on the upper lane*", and then pointed at the lower lane and said "*the child on this side has two wooden blocks, and he or she put them on the lower lane. Right?*". Each child gave a response to the experimenter (e.g., they nodded or said "yes") after hearing the hint. Next, the experimenter introduced the toy car.

> "There are two toy cars on the game board. This car (the experimenter pointed at the Car A on the lower lane which is also the horizontal lane) can only move on this lane (the experimenter pointed at the lower lane at the same time), whereas this car (the experimenter pointed at the Car B on the vertical lane) can only move on that lane (the experimenter pointed at the vertical lane). Now let me move one wooden block from the upper lane to the intersection (the experimenter moved one block from the upper lane to the intersection of the lower lane and the vertical lane). If I moved this car (Car B), the block on the intersection will be returned back (to the upper lane). As a result, there are two blocks on this side (the experimenter pointed at the upper lane) and two blocks on that side (the experimenter pointed at the lower lane). If I move this car (Car A), three blocks will be moved to the black board. The black board is the storehouse; blocks in the storehouse do not belong to anyone. You and other children cannot own the blocks in the storehouse. Hence, there is one block on this side (the experimenter pointed at the upper lane) and no block on that side (the experimenter pointed at the lower lane)."

Finishing the rule introduction, the experimenter moved the three blocks back to the lower lane from the storehouse. Then the experimenter asked the child to move two cars respectively to help them understand the results of restoration and retribution. For example,

> "Now I ask you some questions. Would you like to move this car (the experimenter pointed at Car A)? Try it. (After the child moving the car) How many blocks left on each lane?"

Children could not proceed to the next step until they answered the comprehension questions correctly (i.e., *how many blocks are on the upper and lower lanes*). If children failed to correctly answer the question, the experimenter would reintroduce the rule to the child. In each of the first three experiments, about 80% of children gave the right answer immediately. About 15% of children gave the right answer after the experimenter retelling the rules once again. All children understood the rules after the experimenter reintroducing the rules twice.

1.2 Video watching and comprehension check

Before playing video clips, the experimenter said "*Now I am going to tell you two stories. After I tell you each story, I hope you to judge the children in the story whether their behavior is right or*

wrong.” Then the experimenter introduced children to three emotion cards. In each emotion card there are two emotion faces (see Figure S1).

> “There are 3 emotion cards to help you answer the questions. (The experimenter showed the first card, pointed at the corresponding emotion and said) If you think the character’s behavior is good, point to the ‘happy face’. If you think the character’s behavior is bad, point to the ‘unhappy face’.”
>
> “(Then the experimenter showed the second card and said) And if you think the character’s behavior is a little good, point to the ‘smiling face’. If you think the character’s behavior is very good, point to the ‘grinning face’.”
>
> “(Finally, the experimenter showed the third card) if you think the character’s behavior is a little bad, point to the ‘slightly-frowned face’. If you think the character’s behavior is very bad, point to the ‘tightly-frowned face’.”

Next, the experimenter introduced the two puppets who appeared in the video clip to the child (e.g., *Feng* and *Hua*). Each puppet appeared only once in the experiment. The experimenter said:

> “Let me first introduce you to two kids in the story (The experimenter showed puppet 1). This one named *Feng*. Would you like to say hello to him or her? (The experimenter operated puppet 1 and let the puppet greet the child).”
>
> “(Then the experimenter showed puppet 2) This one named *Hua*. Would you like to say hello to him or her? (The experimenter operated puppet 2 and let the puppet greet the child).”
>
> “These two wooden blocks belong to *Feng* (the experimenter pointing at the blocks on the lower lane), and these two blocks belong to *Hua* (the experimenter pointing at the blocks on the upper lane).”

The experimenter then made sure that the child remembered these puppets. The experimenter pointed at each puppet, and asked the child “what is his or her name”. Children could not proceed to the next step until they answered the correct name of each puppet. If children failed to correctly answer the question, the experimenter would reintroduce two puppets to the child.

The experimenter then played the video clips illustrating the restoration story on an iPad. In one story, *Feng* (the perpetrator) and *Hua* (the restorer) sit at the side of each horizontal lane, facing each other. When *Hua* leaves for the restroom, *Feng* takes a block from *Hua*’s lane, and puts it on his or her own lane (at the intersection of his or her lane and the vertical lane). *Hua* finds it out after he or she coming back and decides to restore the stolen block by moving Car B. As a result, both *Hua* and *Feng* have 2 blocks respectively.

In the other story, *Ho* (the perpetrator) and *Qi* (the punisher) replace Feng and Hua as players. When *Qi* leaves for the restroom, *Ho* takes a block from *Qi*’s lane, and puts it on the intersection of his or her lane and the vertical lane. *Qi* decides to punish *Ho* by moving *Ho*’s blocks to the storehouse by moving Car A.

The experimenter always referred to the puppets using their names rather than “the victim” or “the perpetrator”. The order of the video presentation and the role of the puppets were counterbalanced

across children. A video sample has been uploaded to the Open Science Framework at this weblink https://osf.io/u59kd.

After watching each video clip, the experimenter asked children to repeat what happened in the video. The key points of children's retelling included "*who went to the restroom*", "*who took a block from whom*", "*which car did Hua pushed*", and "*how many blocks each one had in the end*". If they could not repeat the key points correctly, the video clips would be replayed. If children could not answer these questions correctly after replaying three times, the experimenter would finish the test. Table S2 showed how many times the experimenter replayed the video clips for children in three experiments. Results of Chi-square tests showed that, in each of the Experiment 1, 2, and 3, the times of replay in the restoration and punishment conditions did not differ significantly (Experiment 1: χ^2 (2) = 4.69, p = .10; Experiment 2: χ^2 (2) = 3.74, p = .15; Experiment 3: χ^2 (2) = 3.04, p = .22).

1.3 Main evaluation task

After children correctly repeating the story, the experimenter asked them questions on three measures. We presented these questions in the main paper.

1.4 Behavior task

To probe the child's own response to immorality, children were asked to imagine a scenario where their classmate took one of their blocks away when they went to the restroom. Children then asked to move one of the two cars (i.e., either to punish the classmate, or to restore his or her own block).

> "Now you are the player, and these two blocks belong to you (the experimenter pointing at the blocks on the upper lane). Those two blocks belong to one of your classmates (the experimenter pointed at the blocks on the lower lane). You went to the restroom, and your classmate took one of your blocks while you were away. Then you came back from the restroom. You found that one of your blocks was gone, and it was your classmate who took away your block from your lane. Now you can choose to move one of these two cars. Which car do you want to move?"

2 Scripts used in Experiment 4.

The instruction in Experiment 4 is similar to the instruction in the first 3 experiments. The first difference is that all the 3 victims punished the perpetrator, but in different degrees. For example, *Hua* (the mild punisher) punishes *Feng* (the perpetrator) by removing one of *Feng*'s blocks to the trash can. *Ho* (the moderate punisher) punishes *Feng* by removing two of *Feng*'s blocks and *Qi* (the harsh punisher) by removing three blocks. The order of punishers and puppets were counterbalanced before the experiments. After the experimenter told each story, the experimenter asked children to repeat the story. The instruction is the same as in the first 3 experiments. In each story, about 85% of children answered correctly without the experimenter retelling the stories. All the children gave the right answers after retelling the stories for three times (see table S3). The times of repeating the story in mild, moderate, and harsh punishment conditions did not significantly differ, χ^2 (4) = 2.59, p = .63.

After children correctly repeated each story, the experimenter asked them to rate the punishers' behaviors. This part is the same as in the first 3 experiments. The second difference is that we asked children's favorite victim (punisher) instead of liking scores. The experimenter asked children's liking for each puppet ("*which of the 3 punishers do you likes best? Hua, Ho, or Qi?*"). Then the child was given a sticker, and was asked whom to give it to which of the 3 punishers.

The third difference is that we directly asked children what will he or she do when facing the same possession violation as the 3 punishers experienced. Different from the first 3 experiments, children answered more freely instead of choosing between punishment and restoration.

3 Preliminary analyses

We first analyzed whether age and gender affect children's difference scores on behavior ratings or liking scores (using linear model in the package 'lmerTest'; Kuznetsova et al., 2017) and children's sticker allocation (using logistic linear models in the package 'lme4'; Bates et al., 2015). Gender and age are fixed factors in these models. Results revealed that in the first 3 experiments, the effects of age or gender were not significant (*p*s > .05, see table S4). Second, we analyzed whether the video order (play the punishment video or restoration video first) and the times of replaying affect children's behavior ratings or liking scores in the punishment and restoration conditions. Results revealed that in the first 3 experiments, neither the effects of the video order nor times of replaying were significant (*p*s > .05, see table S5 for the restoration condition; see table S6 for the punishment condition).

In Experiment 4, we analyzed whether age and gender affect children's behavior ratings by a mixed linear model (with trials nested within children). Results showed that the effects of age (B = -0.03, SE = 0.04, p = .46), gender (B = 4.27, SE = 4.13, p = .31), or the interaction effects between age and gender (B = -0.06, SE = 0.06, p = .35) were not significant. Then we also used multinomial regression models (using the 'multinom' in the package 'nnet'; Venables & Ripley, 2002) to analyze whether age and gender affect children's answers of favorite punishers and sticker allocations (because children's answers of these two questions are nominal variables which have three levels each). Results revealed no significance of age, gender, and their interactions (*p*s > .05). Thus, we collapsed data across gender and age in the analysis. In addition, we analyzed whether the condition order (mild, moderate, or harsh punishment first) and the times of replaying affect children's behavior ratings. Results revealed no significance of the order of conditions (mild first versus moderate first: B = 0.22, SE = 0.35, p = .53; mild first versus harsh first: B = -0.41, SE = 0.35, p = .25; moderate first versus harsh first: B = -0.63, SE = 0.33, p = .07) and times of repeat (B = -0.12, SE = 0.15, p = .39).

4 Raw data of behavior ratings and liking scores

4.1 Behavior ratings

In each of Experiment 1, 2, and 3, children rated both the perpetrator's and the victim's behavior in 2 conditions (punishment versus restoration). Raw behavior ratings of the perpetrators and the victims (i.e., the punisher or the restorer) are provided in Table S7.

4.2 Liking scores

In Experiment 1, 2, and 3, children answered how much they like the punisher and the restorer. We listed the raw liking scores in Table S8.

5 Children's justification of behavior ratings

After ratings the victims' behavior, children were asked to justify their responses. Justifications were coded using the following categories: *subjective evaluation*, *norm-based*, *state description*, and *Unclassifiable* (see Table S9). A second coder classified all the justifications, and inter-rater reliability was perfect $\kappa = 1.00$.

Table S10 presents the frequencies of children's different justifications in the first 3 experiments. In Experiment 1, more than 95% of justifications were codable; in Experiment 2, 87.5% of justifications were codable. However, compared to those justifications in Experiment 1 and 2, more children in Experiment 3 gave reasons such as *I don't know* or they said nothing (*Unclassifiable*). We compared the number of children whose justifications to the punisher or the restorer belong to *Unclassifiable* type among 3 experiments using Chi-square. As for the justifications to the punisher, the results showed that the number of *others* justifications in Experiment 3 were significantly higher than the number in the first 2 experiments (χ^2 (2) = 12.66, p = .002). As for the justifications to the restorer, the results showed that the number of *others* justifications in Experiment 3 were marginally significantly higher than the number in the first 2 experiments (χ^2 (2) = 5.81, p = .06). It suggests that in Experiment 3, preschoolers found it more difficult to attribute their answers.

We eliminate justifications of the type *Unclassifiable*, then compared the number of children in different categories using Chi-square. More than half of the justifications were *norm-based* or *state description* in all 3 experiments. In Experiment 1, differences were significant among conditions on justification composition to both the punisher (χ^2 (2) = 8.21, p = .02) and the restorer (χ^2 (2) = 6.94, p = .03). Half of the children justified their behavior ratings using *state description*. In Experiment 2, the justification composition to the restorer was significantly differentiated (χ^2 (2) = 15.86, p < .001), and most children used *state description*; but it was marginally significant to the punisher (χ^2 (2) = 5.57, p = .06), which suggested that children tended to used *state description* in justification. In Experiment 3, The results showed that differences were significant among conditions on justification composition to both the punisher (χ^2 (2) = 17.20, p < .001) and the restorer (χ^2 (2) = 12.80, p = .002). About half of the children justified their behavior ratings using *state description*.

We also analyzed whether different types of children (punitive or restorative type, according to their behavior in the behavior task) differentiated in justifications, yet there is no significance of children's type in Experiment 1 (χ^2 (2) = 2.85, p = .24) and Experiment 2 (χ^2 (2) = 2.03, p = .36). Due to the restorative children significantly more than the punitive children, we did not analyze the data in Experiment 3.

6 Data Availability Statement

The datasets ANALYZED for this study can be found in the https://osf.io/2un8d.

7 References

Bates, D., Mächler, M., Bolker, B., & Walker, S. (2015). Fitting linear mixed-effects models using lme4. *Journal of Statistical Software*, *67*(1), 1–48. https://doi.org/10.18637/jss.v067.i01

Kuznetsova, A., Brockhoff, P., & Christensen, R. (2017). lmerTest package: Tests in linear mixed effects models. *Journal of Statistical Software*. https://doi.org/10.18637/JSS.V082.I13

Venables, W. N., & Ripley, B. D. (2002). *Modern Applied Statistics with S* (Fourth). Springer. https://www.stats.ox.ac.uk/pub/MASS4/

8 Supplementary Figures

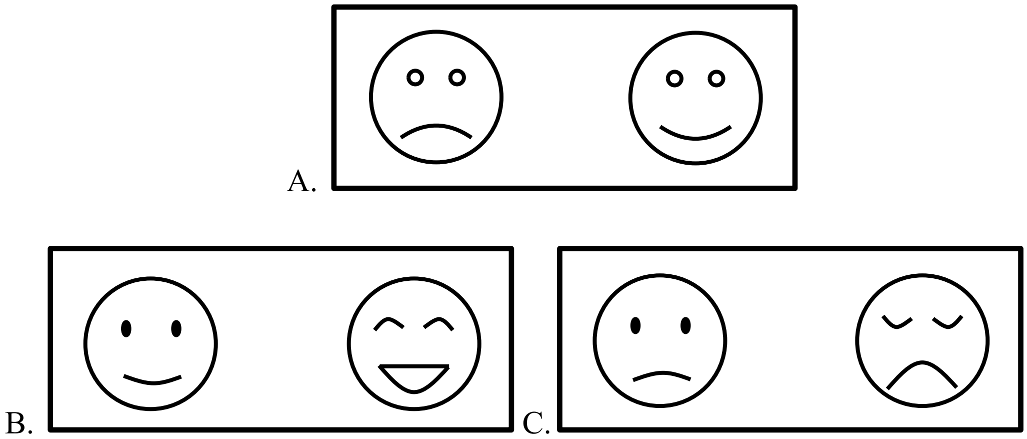

Figure S1. Emotion cards. (A) The happy face and the unhappy face. (B) The smiling face and the grinning face. (C) The slightly-frowned face and the tightly-frowned face. The order of two faces in each card is counterbalanced.

9 Supplementary Tables

Table S1

Number of Participants Passing the Comprehension Questions after Each Time of Retelling the Rules in Experiment 1-3.

Experiment	Times of retelling		
	0 (passed immediately)	1	2
Experiment 1	38	7	3
Experiment 2	39	8	1
Experiment 3	38	7	3

Table S2.

Times of Replaying Video Clips in Experiment 1-3.

Experiment	Condition	Times of replay		
		0 (passed immediately)	1	2-3
Experiment 1	Restoration	32	13	3
	Punishment	41	6	1
Experiment 2	Restoration	34	11	3
	Punishment	34	6	8
Experiment 3	Restoration	24	14	10
	Punishment	27	17	4

Table S3.

Times of Repeating the stories in Experiment 4.

Condition	Times of repeat		
	0(passed immediately)	1	2-3
Mild punishment	35	4	1
Moderate punishment	34	6	0
Harsh punishment	34	4	2

Table S4

The Effects of Age and Gender on children's performance in Experiment 1-3.

		Experiment 1			Experiment 2			Experiment 3		
		Age	Gender	Age* Gender	Age	Gender	Age* Gender	Age	Gender	Age* Gender
Difference scores on behavior ratings	*B*	0.04	-1.83	0.02	-0.09	-6.81	0.11	<.001	-4.33	0.07
	SE	0.05	4.87	0.08	0.04	4.39	0.07	0.05	4.34	0.07
	p	0.47	0.71	0.79	0.06	0.13	0.12	0.97	0.33	0.31
Difference scores on liking scores	*B*	-0.08	-2.41	0.04	0.07	-0.64	0.01	-0.03	-5.87	0.09
	SE	0.06	6.01	0.1	0.07	6.87	0.11	0.06	5.43	0.09
	p	0.2	0.69	0.68	0.31	0.93	0.94	0.6	0.29	0.3
Sticker allocation	*B*	0.15	10.25	-0.16	-0.05	-13.55	0.21	0.15	12.44	-0.22
	SE	0.11	10.09	0.16	0.1	10.24	0.16	0.11	10.34	0.17
	p	0.18	0.31	0.32	0.59	0.19	0.18	0.19	0.23	0.19

note. Age*Gender means the interaction effect of age and gender.

Table S5

The Effects of the Video Order and Times of Replay on children's Behavior ratings and Liking scores in Restoration Condition, Experiment 1-3.

		Experiment 1		Experiment 2		Experiment 3	
		Behavior ratings	Liking scores	Behavior ratings	Liking scores	Behavior ratings	Liking scores
Times of replay	*B*	-0.02	-0.03	0.13	0.16	0.12	-0.22
	SE	0.22	0.17	0.18	0.25	0.17	0.18
	p	.93	.87	.49	.51	.48	.23
The order of the video (Punishment or restoration first)	*B*	-0.07	-0.35	-0.09	-0.02	0.18	0.001
	SE	0.31	0.24	0.22	0.29	0.31	0.31
	p	.83	.15	.68	.95	.55	.99
R^2		.002	.07	.01	.01	.03	.04
Adjusted R^2		-.04	.02	-.03	-.03	-.01	-.01

Table S6

The Effects of the Video Order and Times of Replay on children's Behavior ratings and Liking scores in Punishment Condition, Experiment 1-3.

		Experiment 1		Experiment 2		Experiment 3	
		Behavior ratings	Liking scores	Behavior ratings	Liking scores	Behavior ratings	Liking scores
Times of replay	*B*	-0.26	0.31	0.157	-0.14	0.31	0.28
	SE	0.38	0.37	0.188	0.16	0.20	0.19
	p	.51	.41	.41	.37	.13	.16
The order of the video (Punishment or restoration first)	*B*	-0.19	-0.05	-0.01	0.16	0.03	0.17
	SE	0.33	0.32	0.31	0.26	0.28	0.27
	p	.58	.89	.97	.47	.92	.54
R^2		.02	.02	.02	.04	.05	.05
Adjusted R^2		-.03	-.03	-.03	-.003	.01	.01

Table S7

Means (Standard Deviation) of Behavioral Ratings of the Perpetrators and the Victims in Experiment 1-3 as a Function of Conditions (Punishment versus Restoration)

Experiment	Character	Behavior ratings		
		Punishment	Restoration	Total
Experiment 1	Perpetrator	1.38 (.49)	1.48 (.55)	1.43 (.52)
	Victim	2.92 (1.11)	3.40 (.89)	3.16 (1.03)
Experiment 2	Perpetrator	1.44 (.62)	1.50 (.62)	1.47 (.61)
	Victim	3.08 (1.01)	3.60 (.71)	3.34 (.90)
Experiment 3	Perpetrator	1.44 (.62)	1.42 (.61)	1.45 (.61)
	Victim	3.02 (.98)	3.17 (.98)	3.09 (.97)

Table S8

Means (Standard Deviation) of Liking Scores of the Punisher and the Restorer in Experiment 1-3.

Experiment	Punisher	Restorer
Experiment 1	3.17 (1.08)	3.56 (.71)
Experiment 2	3.45 (.85)	3.31 (.95)
Experiment 2	3.17 (.95)	3.33 (1.00)

Table S9

Coding Scheme of Children's Reasoning about Their Responses to the Punisher and Restorer

Category	Description	Examples
Subjective evaluation	Explicit references to others' morality or intention, to fulfilling their own desires, or to voicing their own needs.	"*He is a bad boy*"; "*I just want to do that*".
Norm-based	Explicit references to a standard of being right or wrong, fair or unfair, good or bad.	"*It is impolite to take other's blocks without permission*"; "*we are equal in this way*".
State description	Explicit references to the scenario that was already happened, or to repeating what the perpetrator or victims has remarked in the video clips.	"*Hua has moved the blocks to the storehouse by pushing this car*"; "*This car moves in this lane*".
Unclassifiable	Justifications that could not be coded into the other categories.	"*I don't know*"; children did not give any responses.

Table S10

Frequencies of Different Types of Justifications in Experiment 1-3.

Victim	Justification type	Experiment		
		Experiment 1	Experiment 2	Experiment 3
Punisher	Subjective evaluation	8	7	4
	Norm-based	15	16	8
	State description	24	19	23
	Unclassifiable	1	6	13
Restorer	Subjective evaluation	13	4	8
	Norm-based	10	13	8
	State description	24	25	24
	Unclassifiable	1	6	8
